# Diagnostic Performance of CMR, SPECT, and PET Imaging for the Identification of Coronary Artery Disease: A Meta-Analysis

**DOI:** 10.3389/fcvm.2021.621389

**Published:** 2021-05-07

**Authors:** Jianfeng Xu, Fei Cai, Changran Geng, Zheng Wang, Xiaobin Tang

**Affiliations:** ^1^Department of Nuclear Sciences and Engineering, Nanjing University of Aeronautics and Astronautics, Nanjing, China; ^2^JYAMS PET Research and Development Limited, Nanjing, China

**Keywords:** myocardial perfusion imaging, coronary artery disease, diagnostic performance, meta-analysis, non-invasive modality

## Abstract

**Background:** Myocardial perfusion imaging modalities, such as cardiac magnetic resonance (CMR), single-photon emission computed tomography (SPECT), and positron emission tomography (PET), are well-established non-invasive diagnostic methods to detect hemodynamically significant coronary artery disease (CAD). The aim of this meta-analysis is to compare CMR, SPECT, and PET in the diagnosis of CAD and to provide evidence for further research and clinical decision-making.

**Methods:** PubMed, Web of Science, EMBASE, and Cochrane Library were searched. Studies that used CMR, SPECT, and/or PET for the diagnosis of CAD were included. Pooled sensitivity, specificity, positive likelihood ratio, negative likelihood ratio, diagnostic odds ratio with their respective 95% confidence interval, and the area under the summary receiver operating characteristic (SROC) curve were calculated.

**Results:** A total of 203 articles were identified for inclusion in this meta-analysis. The pooled sensitivity values of CMR, SPECT, and PET were 0.86, 0.83, and 0.85, respectively. Their respective overall specificity values were 0.83, 0.77, and 0.86. Results in subgroup analysis of the performance of SPECT with ^201^Tl showed the highest pooled sensitivity [0.85 (0.82, 0.88)] and specificity [0.80 (0.75, 0.83)]. ^99m^Tc-tetrofosmin had the lowest sensitivity [0.76 (0.67, 0.82)]. In the subgroup analysis of PET tracers, results indicated that ^13^N had the lowest pooled sensitivity [0.83 (0.74, 0.89)], and the specificity was the highest [0.91 (0.81, 0.96)].

**Conclusion:** Our meta-analysis indicates that CMR and PET present better diagnostic performance for the detection of CAD as compared with SPECT.

## Introduction

Coronary artery disease (CAD) is one of the main causes of mortality worldwide, which is mainly attributed to coronary artery atherosclerosis (1). It is noteworthy that the burden of atherosclerotic cardiovascular disease in low- and middle-income countries is still increasing (2). Based on Global Burden of Disease program, statistics revealed that the global prevalence of CAD had reached ~154 million in the year 2016 (3). The overriding goal of common treatment approaches is to relieve symptom through drug therapy and vascular remodeling and to avoid potential cardiovascular events in the future (4–7).

At present, it is important to assess the degree of coronary artery stenosis accurately, so as to provide the basis for the upcoming treatment. Fractional flow reserve (FFR) has been regarded as a mature and precise approach to evaluate the hemodynamic relevance of a coronary artery stenosis; nevertheless, FFR is also an invasive approach (8). Non-invasive myocardial perfusion imaging (MPI) modalities, such as cardiac magnetic resonance (CMR), single-photon emission computed tomography (SPECT), and positron emission tomography (PET), are well-established methods for the detection of hemodynamically significant CAD (9–12). CMR characterized by high spatial resolution images of myocardial perfusion has been verified to be of assistance in guiding patients with CAD (13–15). On the other hand, SPECT is the most commonly performed diagnostic method in patients with stable coronary heart disease. Although SPECT has been reported that the sensitivity of detection of high-risk subgroups is not sounding, there are certain inaccuracies in screening patients for invasive coronary angiography (CA) (16–18). Common SPECT radiotracers include ^99m^Tc-sestamibi (^99m^Tc-MIBI), ^99m^Tc-tetrofosmin, and ^201^Tl (19–21). PET is also a widely used MPI modality for the detection of hemodynamic significance in CAD. It offers better diagnostic performance, improved resolution of images, and inherent attenuation correction (22–24). Furthermore, compared with SPECT protocol, PET imaging provides lower radiation exposure to patients due to the physical property of radiotracers including ^82^Rb, ^13^N-ammonia, and ^15^O-H_2_O (25– 27).

To our knowledge, accumulated studies and meta-analysis have evaluated diagnostic performance of both invasive and non-invasive approaches for the confirmation of CAD (28–34). The aim of this meta-analysis was to generate a more comprehensive comparison of CMR, SPECT, and PET in the detection of CAD by collating the available evidence and subsequently to provide meaningful and hints for not only the field of implement research but also for the implementation and decision-making in clinical settings.

## Materials and Methods

Each process of this meta-analysis was conducted based on preferred reporting items for systematic reviews and meta-analysis (PRISMA) (35).

### Search Strategy and Selection Criteria

We conducted a systematic search of the electronic databases, PubMed, Web of Science, EMBASE, and Cochrane Library from inception to July 31, 2020, with articles in English language considered. The following key terms were used for the database research: “cardiac magnetic resonance,” “CMR,” “single-photon emission computed tomography,” “SPECT,” “positron emission tomography,” “PET,” “myocardial perfusion imaging,” “MPI,” and “coronary artery disease.” The bibliographies of these articles were also screened for any eligible studies. Inclusion criteria were as follows: (1) CMR, SPECT, and/or PET were used for the diagnosis of CAD in patients with suspected or confirmed CAD; (2) either CA or FFR was referred as the gold standard to assess diagnostic performance; (3) absolute numbers of participants with true positive (TP), false positive (FP), true negative (TN), and false negative (FN) outcomes could be excerpted directly in the original article or calculated on the basis of information presented in the literature. If studies were conducted by the same group, those with the largest sample size or the most sufficient information were enrolled. Articles were excluded if they were case report, review, letters, news, conference abstract, animal study, or studies without the necessary variables mentioned above.

Two independent investigators (Jianfeng Xu and Fei Cai) conducted the process of literature search and study inclusion. Discrepancies were resolved by discussion. If no consensus was reached, a third author (Changran Geng) was involved.

### Data Extraction and Quality Assessments

Two reviewers (Zheng Wang and Xiaobin Tang) independently performed the title and abstract screening according to the inclusion criteria. Full-text reading of the literature was conducted for the final inclusion. The following information was extracted from each study: first author's name, year of publication, number of patients analyzed, reference standard (CA or FFR), level of analysis (patient-based or vessel-based), MPI modality and radiotracers used in the study, and absolute number of patients with TP, TN, FP, and FN results.

To assess the quality of the included studies, we used the Quality Assessment of Diagnostic Accuracy Studies-2 (QUADAS-2) criteria. This method contains components in terms of participant selection, index test, and reference standard, as well as flow and timing (36).

### Statistical Analysis

The Stata 15.0 software and Review Manager 5.3 software were employed for all statistical analyses at the study level. *p* < 0.05 was considered to be of statistical significance. We calculated pooled sensitivity, specificity, positive likelihood ratio, negative likelihood ratio, diagnostic odds ratio, and the 95% confidence intervals and the area under the summary receiver operating characteristic curve (AUC). We used Cochran *Q* and the *I*^2^ statistics to detect the heterogeneity of studies included. *I*^2^ values of 0–25%, 25–50%, 50–75%, and 75–100% indicated insignificant, low, moderate, and high heterogeneity, respectively (37). Metaregression was performed to explore the possible source of heterogeneity between the included studies. A Hanley and McNeil method was used to assess for potential differences in AUCs. χ^2^ tests were employed to compare the differences in sensitivities and specificities using the bivariate model. We created funnel plots to assess potential bias of publication. Deeks' method was used to statistically check the asymmetry of the funnel plot and detect publication bias. We conducted sensitivity analysis to evaluate the impacts of single study on the overall outcomes.

## Results

### Study Selection and Characteristics

**Figure 1:**
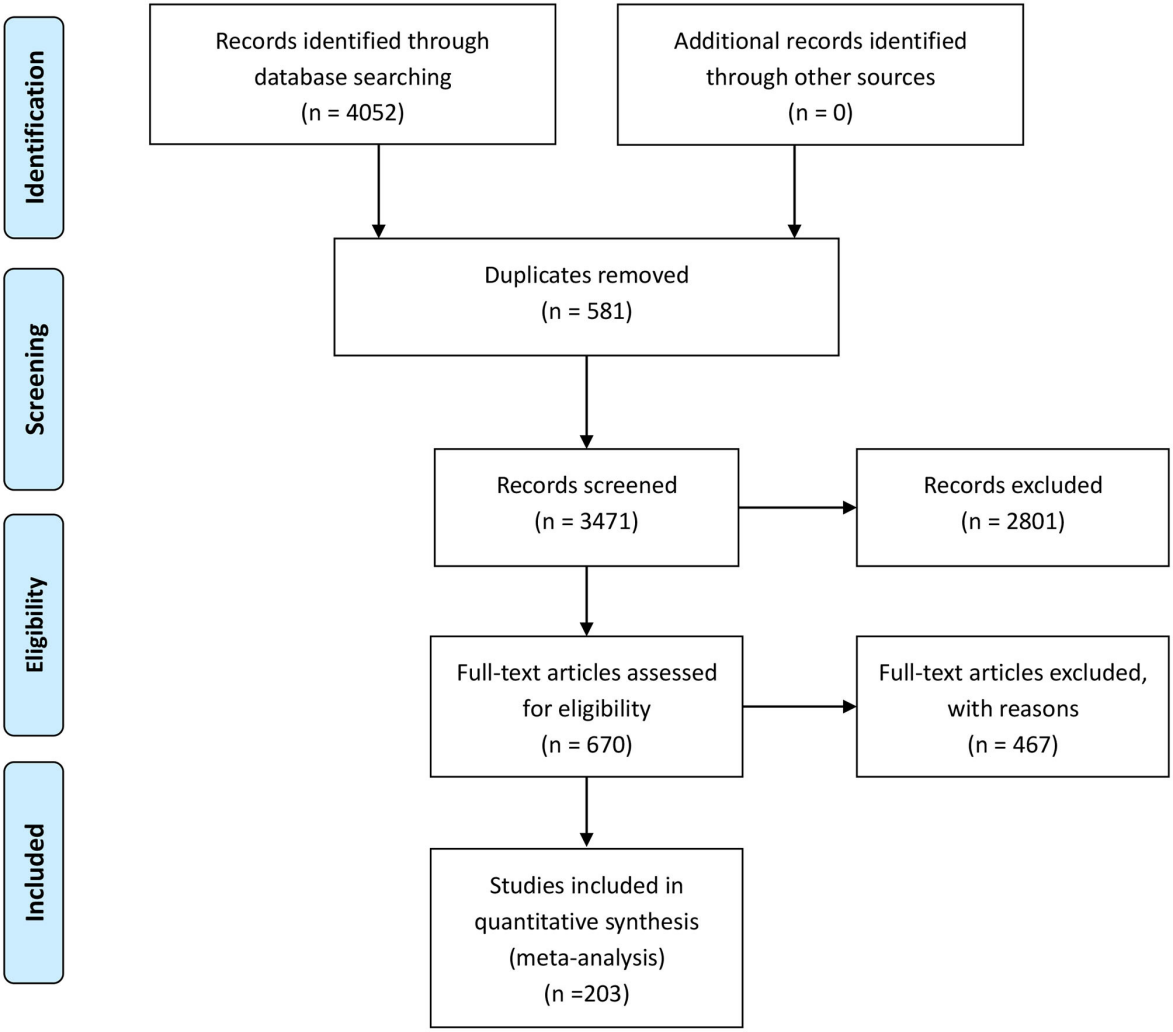
Search results and flowchart of the meta-analysis.

**Figure 2:**
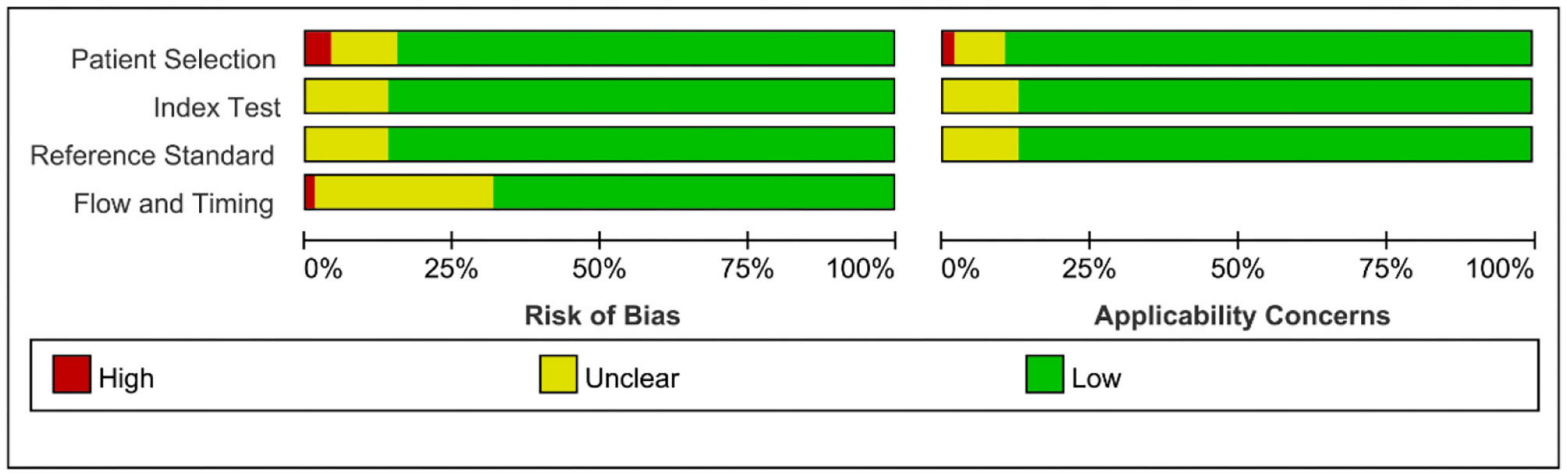
Risk of bias and applicability concerns on the QUADAS-2 tool of the included studies.

A total of 4,052 articles were identified from the databases searched. Among them, 581 duplicates were removed, and 2,801 studies were excluded through an initial screening. After a full text assessment for eligibility of the remaining 670 articles, 203 articles with 215 studies, 23,942 patients, and 20,213 artery territories were identified for inclusion in this meta-analysis. No additional studies were found through reference screening of the included articles. Figure 1 shows the flow of the database search and literature selection process. The results of quality evaluation of the included studies manifested that the high quality of the included studies (see Figure 2).

### Diagnostic Performance of MPI Modalities

**Figure 3:**
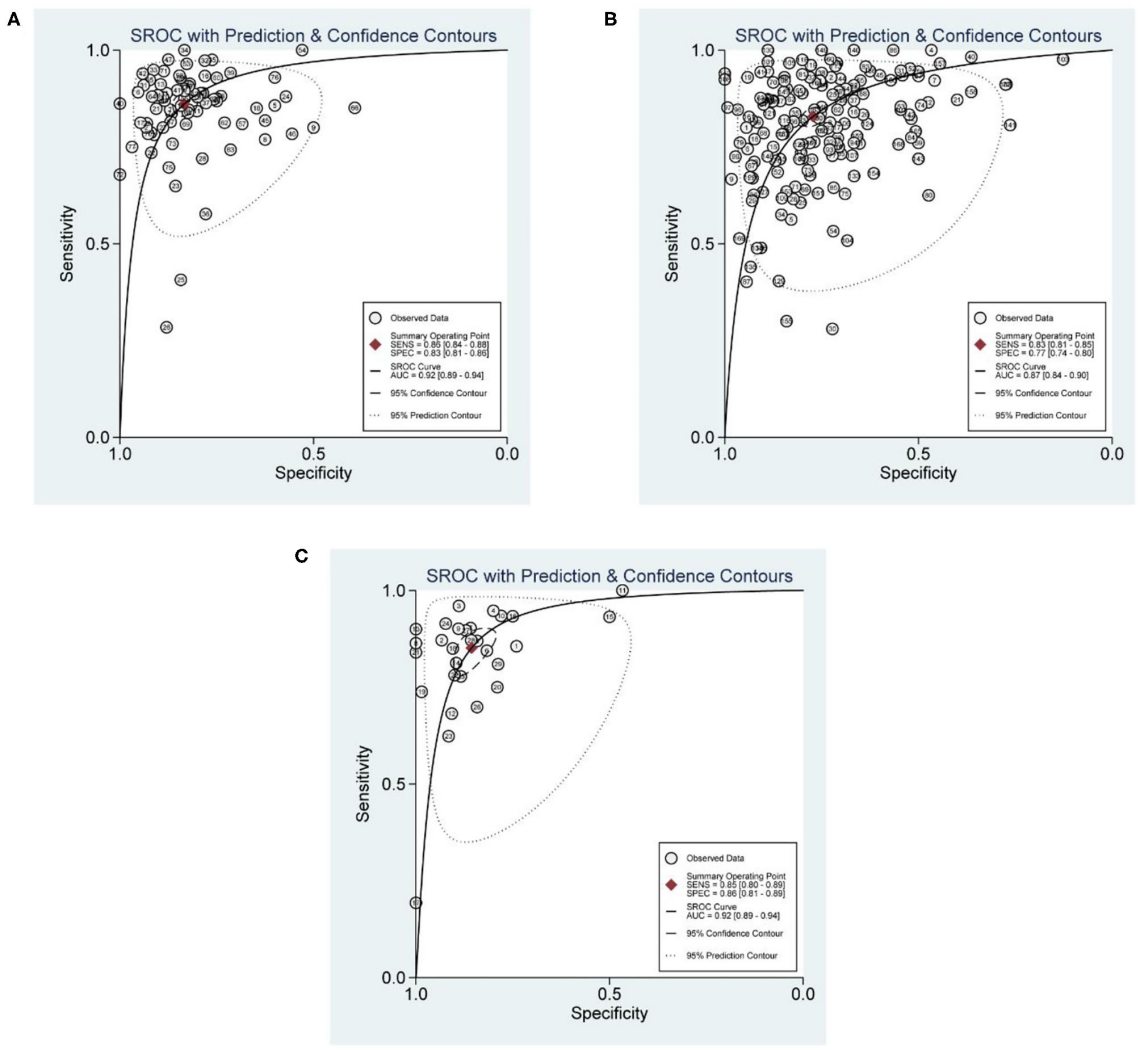
SROC curves for diagnostic performance of CMR, SPECT, and PET. **(A)** SROC curve for diagnostic performance of CMR. **(B)** SROC curves for diagnostic performance of SPECT. **(C)** SROC curves for diagnostic performance of PET.

The numbers of articles included in the analysis of CMR, SPECT, and PET were 56, 134, and 25, respectively. The pooled sensitivities of CMR, SPECT, and PET were 0.86 (0.84, 0.88), 0.83 (0.81, 0.85), and 0.85 (0.80, 0.89), respectively (*p* = 0.109). The overall specificities were 0.83 (0.81, 0.86), 0.77 (0.74, 0.80), and 0.86 (0.81, 0.89) for CMR, SPECT, and PET, respectively (SPECT vs. PET, *p* < 0.01; SPECT vs. CMR, *p* < 0.01; PET vs. CMR, *p* = 0.716). The AUC values of CMR, SPECT, and PET were 0.92 (0.89, 0.94), 0.87 (0.84, 0.90), and 0.92 (0.89, 0.94) (SPECT vs. PET, *p* < 0.01; SPECT vs. CMR, *p* < 0.01; PET vs. CMR, *p* = 0.215) (Table 1 and Figure 3). Two studies (Dekker et al. and Driessen et al.) reported evaluation of quantitative myocardial blood flow (MBF), and results showed that MBF was lower in patients with CAD than that in the non-CAD participants in both studies.

**Table 1:** Diagnostic performance of MPI modalities.

**Modalities**	**Sensitivity**				**Specificity**				**+LR**	**–LR**	**DOR**	**SROC curve AUC**
	**Sensitivity**	***I*^**2**^ (%)**	***Q* value**	***P***	**Specificity**	***I*^**2**^ (%)**	***Q* value**	***p***				
PET	0.85 [0.80, 0.89]	90.24 [87.55, 92.93]	286.87	<0.01	0.86 [0.81, 0.89]	77.88 [70.19, 85.58]	126.61	<0.01	5.9 [4.6, 7.7]	0.17 [0.13, 0.23]	34 [25, 47]	0.92 [0.89, 0.94]
SPECT	0.83 [0.81, 0.85]	92.81 [92.07, 93.55]	2350.03	<0.01	0.77 [0.74, 0.80]	93.69 [93.07, 94.32]	2679.42	<0.01	3.6 [3.3, 4.1]	0.22 [0.20, 0.25]	16 [14, 19]	0.87 [0.84, 0.90]
CMR	0.86 [0.84, 0.88]	84.53 [81.56, 87.50]	491.31	<0.01	0.83 [0.81, 0.86]	84.29 [81.26, 87.32]	483.76	<0.01	5.2 [4.5, 6.0]	0.17 [0.14, 0.20]	31[24, 40]	0.92 [0.89, 0.94]

*CMR, cardiac magnetic resonance; SPECT, single-photon emission computed tomography; PET, positron emission tomography; MPI, myocardial perfusion imaging; +LR, positive likelihood ratio; –LR, negative likelihood ratio; DOR, diagnostic odds ratio; SROC, summary receiver operating characteristic; AUC, area under the SROC curve*.

### Subgroup Analysis of the Performance of CMR

With regard to the reference standard, the overall sensitivity of CMR was 0.87 (0.83, 0.89) when CA was the reference standard, it yielded a specificity of 0.82 (0.79, 0.85), and the AUC value was 0.91 (0.88, 0.93). When FFR was used as the reference standard, the pooled sensitivity and specificity were 0.85 (0.81, 0.88) and 0.86 (0.81, 0.89), with an AUC value of 0.91 (0.88, 0.93). The pooled sensitivity was higher at the patient level [0.88 (0.86, 0.90)] than that at the vessel level [0.83 (0.78, 0.86)]. The pooled specificities at the patient level and the territory level were 0.81 (0.78, 0.84) and 0.86 (0.82, 0.90), respectively. The AUCs were equal at the two levels (Table 2). The pooled sensitivity and specificity were 0.89 (0.85, 0.91) and 0.80 (0.76, 0.83) in prospective CMR studies. Results of subgroup analyses (type of study, data assessment, prevalence of CAD and multivessel disease, and patient selection) for the diagnostic performance of CMR on patient-based level are listed in Supplementary Table 4.

**Table 2:** Subgroup analysis of CMR MPI.

	**Sensitivity**				**Specificity**				**+LR**	**–LR**	**DOR**	**SROC curve AUC**
	**Sensitivity**	***I*^**2**^ (%)**	***Q* value**	***P***	**Specificity**	***I*^**2**^ (%)**	***Q* value**	***p***			
**Reference standard**												
Coronary angiography	0.87 [0.83, 0.89]	88.27 [85.74, 90.81]	434.95	<0.01	0.82 [0.79, 0.85]	81.87 [77.43, 86.31]	281.30	<0.01	4.8 [4.1, 5.7]	0.16 [0.13, 0.20]	30 [22, 40]	0.91 [0.88, 0.93]
FFR	0.85 [0.81, 0.88]	62.97 [47.05, 78.89]	64.82	<0.01	0.86 [0.81, 0.89]	84.86 [79.74, 89.98]	158.50	<0.01	5.9 [4.4, 8.0]	0.18 [0.14, 0.22]	33 [22, 51]	0.91 [0.88, 0.93]
**Analytic level**												
Artery based	0.83 [0.78, 0.86]	87.64 [84.11, 91.17]	242.73	<0.01	0.86 [0.82, 0.90]	90.23 [87.63, 92.83]	307.08	<0.01	6.0 [4.5, 7.9]	0.20 [0.16, 0.26]	29 [20, 44]	0.91 [0.88, 0.93]
Patient based	0.88 [0.86, 0.90]	75.46 [68.51, 82.41]	183.38	<0.01	0.81 [0.78, 0.84]	66.85 [56.67, 77.04]	135.75	<0.01	4.6 [3.9, 5.4]	0.15 [0.12, 0.18]	31 [23, 42]	0.91 [0.88, 0.93]

*CMR, cardiac magnetic resonance; FFR, fractional flow reserve; MPI, myocardial perfusion imaging; +LR, positive likelihood ratio; –LR, negative likelihood ratio; DOR, diagnostic odds ratio; SROC, summary receiver operating characteristic; AUC, area under the SROC curve*.

### Subgroup Analysis of the Performance of SPECT

The numbers of studies using ^99m^Tc-tetrofosmin, ^99m^Tc-MIBI, and ^201^Tl for SPECT tracers were 21, 53, and 40, respectively. Studies that used two or more tracers were not rerolled in this pooled analysis. Results manifested that ^201^Tl showed the highest pooled sensitivity [0.85 (0.82, 0.88)] and specificity [0.80 (0.75, 0.83)]. ^99m^Tc-tetrofosmin had the lowest sensitivity [0.76 (0.67, 0.82)]. The pooled specificity of ^99m^Tc-MIBI was the lowest [0.76 (0.71, 0.80)]. The AUC values of ^99m^Tc-tetrofosmin, ^99m^Tc-MIBI, ^201^Tl were 0.85 (0.81, 0.88), 0.87 (0.8, 0.90), and 0.90 (0.87, 0.92), respectively. Analysis based on reference standard (CA or FFR) showed that the sensitivity was higher but the specificity was lower using CA as reference. Besides, analysis on the analytic level presented higher sensitivity at the patient level and higher specificity at the artery territory level. See more details in Table 3. The pooled sensitivity and specificity were 0.88 (0.86, 0.90) and 0.73 (0.69, 0.77) in prospective SPECT studies. Results of subgroup analyses (type of study, data assessment, prevalence of CAD and multivessel disease, and patient selection) for the diagnostic performance of SPECT are listed in Supplementary Table 5.

**Table 3:** Subgroup analysis of SEPCT MPI.

	**Sensitivity**				**Specificity**				**+LR**	**–LR**	**DOR**	**SROC curve AUC**
	**Sensitivity**	***I*^**2**^ (%)**	***Q* value**	***P***	**Specificity**	***I*^**2**^ (%)**	***Q* value**	***p***			
**Tracers**												
^99m^Tc-MIBI	0.84 [0.81, 0.87]	92.00 [90.63, 93.36]	812.13	<0.01	0.76 [0.71, 0.80]	88.95 [86.87, 91.03]	588.34	<0.01	3.5 [2.9, 4.1]	0.21 [0.17, 0.25]	17 [13, 22]	0.87 [0.84, 0.90]
^99m^Tc-tetrofosmin	0.76 [0.67, 0.82]	91.56 [89.05, 94.08]	260.79	<0.01	0.80 [0.72, 0.86]	88.79 [85.16, 92.42]	196.26	<0.01	3.8 [2.8, 5.1]	0.30 [0.23, 0.40]	12 [8, 19]	0.85 [0.81, 0.88]
^201^Tl	0.85 [0.82, 0.88]	84.34 [80.66, 88.02]	325.69	<0.01	0.80 [0.75, 0.83]	88.04 [85.45, 90.64]	426.56	<0.01	4.2 [3.5, 5.1]	0.19 [0.16, 0.22]	22 [17, 29]	0.90 [0.87, 0.92]
**Reference standard**												
Coronary angiography	0.84 [0.82, 0.86]	92.88 [92.10, 93.65]	2091.89	<0.01	0.77 [0.74, 0.79]	93.74 [93.08, 94.40]	2379.50	<0.01	3.6 [3.2, 4.0]	0.21 [0.19, 0.24]	17 [14, 20]	0.87 [0.84, 0.90]
FFR	0.74 [0.66, 0.81]	90.82 [87.81, 93.83]	206.95	<0.01	0.82 [0.74, 0.87]	90.99 [88.05, 93.93]	210.83	<0.01	4.1 [2.9, 5.6]	0.31 [0.24, 0.42]	13 [8, 20]	0.85 [0.82, 0.88]
**Analytic level**												
Artery based	0.71 [0.68, 0.74]	86.76 [83.61, 89.92]	339.94	<0.01	0.84 [0.81, 0.86]	90.91 [88.98, 92.85]	495.23	<0.01	4.4 [3.6, 5.3]	0.35 [0.31, 0.39]	13 [10, 16]	0.83 [0.80, 0.86]
Patient based	0.86 [0.84, 0.88]	91.36 [90.27, 92.46]	1424.34	<0.01	0.74 [0.70, 0.77]	92.09 [91.11, 93.07]	1555.84	<0.01	3.3 [2.9, 3.8]	0.18 [0.16, 0.21]	18 [15, 22]	0.88 [0.85, 0.91]

*SPECT, single-photon emission computed tomography; FFR, fractional flow reserve; MPI, myocardial perfusion imaging; +LR, positive likelihood ratio; –LR, negative likelihood ratio; DOR, diagnostic odds ratio; SROC, summary receiver operating characteristic; AUC, area under the SROC curve*.

### Subgroup Analysis of the Performance of PET

The numbers of studies utilizing ^82^Rb, ^13^N-ammonia, and ^15^O for PET tracers were 15, 5, and 3, respectively. One study that used ^62^Cu was not included in this pooled analysis. ^82^Rb demonstrated the highest pooled sensitivity [0.87 (0.79, 0.93)]. Although ^13^N showed the lowest pooled sensitivity [0.83 (0.74, 0.89)], its pooled specificity was the highest [0.91 (0.81, 0.96)]. The AUC values of ^82^Rb, ^13^N-ammonia, and ^15^O were 0.91 (0.89, 0.94), 0.92 (0.89, 0.94), and 0.88 (0.85, 0.91), respectively. Moreover, analysis on the basis of reference standard showed that the specificity was higher using CA as reference. Besides, with respect to analytic level, the pooled analysis yielded higher sensitivity at the patient level (Table 4). In prospective PET studies, the pooled sensitivity and specificity were 0.88 (0.74, 0.95) and 0.88 (0.66, 0.96). Results of subgroup analyses (type of study, data assessment, prevalence of CAD and multivessel disease, and patient selection) for the diagnostic performance of PET are listed in Supplementary Table 6.

**Table 4:** Subgroup analysis of PET MPI.

	**Sensitivity**				**Specificity**				**+LR**	**–LR**	**DOR**	**SROC curve AUC**
	**Sensitivity**	***I*^**2**^ (%)**	***Q* value**	***P***	**Specificity**	***I*^**2**^ (%)**	***Q* value**	***p***			
**Tracers**												
^13^N	0.83 [0.74, 0.89]	74.92 [52.27, 97.57]	15.95	<0.01	0.91 [0.81, 0.96]	91.03 [84.79, 97.27]	44.59	<0.01	8.8 [4.2, 18.2]	0.19 [0.13, 0.29]	46 [21, 100]	0.92 [0.89, 0.94]
^15^O	0.84 [0.77, 0.89]	59.22 [14.41, 100.00]	7.36	0.06	0.81 [0.77, 0.85]	35.09 [0.00, 100.00]	4.62	0.20	4.5 [3.5, 5.8]	0.20 [0.14, 0.29]	23 [12, 41]	0.88 [0.85, 0.91]
^82^Rb	0.87 [0.79, 0.93]	93.47 [91.42, 95.51]	260.22	<0.01	0.84 [0.77, 0.89]	74.30 [62.42, 86.19]	66.16	<0.01	5.4 [4.0, 7.2]	0.15 [0.09, 0.24]	36 [24, 53]	0.91 [0.89, 0.94]
**Reference standard**												
Coronary angiography	0.85 [0.77, 0.91]	93.00 [90.76, 95.24]	242.75	<0.01	0.86 [0.81, 0.90]	57.52 [35.25, 79.78]	40.02	<0.01	6.1 [4.8, 7.8]	0.17 [0.11, 0.26]	35 [24, 53]	0.91 [0.89, 0.94]
FFR	0.85 [0.79, 0.89]	69.23 [50.03, 88.42]	32.50	<0.01	0.84 [0.76, 0.90]	87.73 [81.74, 93.73]	81.52	<0.01	5.5 [3.6, 8.3]	0.18 [0.13, 0.24]	31 [19, 48]	0.91 [0.88, 0.93]
**Analytic level**												
Artery based	0.80 [0.72, 0.86]	76.27 [59.86, 92.69]	29.50	<0.01	0.86 [0.82, 0.89]	56.81 [22.77, 90.85]	16.21	0.02	5.7 [4.3, 7.4]	0.23 [0.17, 0.33]	24 [15, 39]	0.90 [0.87, 0.92]
Patient based	0.87 [0.80, 0.91]	92.28 [89.93, 94.63]	259.05	<0.01	0.86 [0.78, 0.91]	80.28 [72.44, 88.13]	101.44	<0.01	6.1 [4.1, 9.2]	0.15 [0.11, 0.22]	40 [27, 60]	0.93 [0.90, 0.95]

*PET, positron emission tomography; FFR, fractional flow reserve; MPI, myocardial perfusion imaging; +LR, positive likelihood ratio; –LR, negative likelihood ratio; DOR, diagnostic odds ratio; SROC, summary receiver operating characteristic; AUC, area under the SROC curve*.

### Heterogeneity and Publication Bias

Deeks' tests for publication bias yielded *p* values of 0.81, <0.01, and 0.13 for CMR, SPECT, and PET, which revealed that there was possible publication bias in the analysis of SPECT.

### Sensitivity Analysis

We performed the sensitivity analysis to assess the impacts of a single study on the overall outcomes. No outlier of CMR, SPECT, or PET was identified.

## Discussion

CAD has become the primary cause of deaths worldwide (38). CA and FFR are commonly regarded as the reference standard for the detection and evaluation of prognosis of CAD. However, they are an invasive approach and have potential risks for human body (28, 39). In contrast, non-invasive techniques such as CMR, SPECT, and PET embraced higher spatial resolution and low dose of radiation (CMR is free of nuclear radiation); they are undergoing an increase in clinical utility despite the relatively high cost (12, 40, 41). Nowadays, they remain to be the most reliable approaches for diagnosis of CAD with hemodynamic significance and guide the choice of treatment and assessment prognosis (27).

We conducted a meta-analysis to estimate the performance of CMR, SPECT, and PET for the diagnosis of obstructive CAD. The analysis was based on study design, type of analysis performed in the individual studies, type of radiotracers for SPECT and PET, reference standard, analysis level, and patient selection, some of which have not been discussed in previous meta-analyses (27, 29, 42). This is one of the strengths of this study. Results revealed that CMR, SPECT, and PET presented high sensitivity and specificity for the detection of CAD. There were no statistically significant differences in sensitivities between CMR, SPECT, and PET (*p* = 0.109). The differences in specificities were statistically significant (SPECT vs. PET, *p* < 0.01; SPECT vs. CMR, *p* < 0.01; PET vs. CMR, *p* = 0.716). The AUC values of CMR, SPECT, and PET indicated that CMR and PET showed better diagnostic performance for the detection of CAD as compared with SPECT (SPECT vs. PET, *p* < 0.01; SPECT vs. CMR, *p* < 0.01; PET vs. CMR, *p* = 0.215). Results manifested that ^201^Tl showed the highest pooled sensitivity [0.85 (0.82, 0.88), *p* < 0.01] and specificity [0.80 (0.75, 0.83), *p* < 0.01]. ^13^N revealed the biggest AUC [0.92 (0.89, 0.94), *p* = 0.087]. For the three modalities, when CA was the reference standard, the overall sensitivity of CMR was higher, but the specificity was lower. Besides, the pooled analysis yielded higher sensitivity at the patient level than vessel level. The diagnostic performance in this analysis was not quite similar to previous meta-analyses (27, 29). In this study, when FFR was utilized as the reference standard, the AUCs for both CMR and PET were equal. The reason may be the different source of heterogeneity between studies included. Although other imaging techniques such as echocardiography and CT have been proven sensitive in previous studies, in consideration of the aim of this study and the considerable amount of articles included, we did not consider including the two modalities (43–45).

In this meta-analysis, we did a detailed literature retrieval to enhance the probability of searching as much related studies as we can. Two independent reviewers performed the entire process of data extraction using a standardized spreadsheet. Furthermore, we evaluated the heterogeneity between the studies included. There were significant heterogeneities among studies. Distinctions in the year of publication, study methodology and patient characteristics, and reference standard may contribute to the heterogeneity. Metaregression was performed to investigate the likely cause of heterogeneity. As was shown in the metaregression, for CMR, year of publication, reference standard, and analytic basis (patient or vessel based) were the possible source of heterogeneity. For the analysis of SPECT and PET, the source of heterogeneity may be attributed to the type of radiotracers, reference standard, and analytic basis. The inclusion of covariates in this meta-analysis was based on the information extracted from the included articles, and the potential role of covariates in scientific and clinical application was also considered. Accordingly, not all potential sources of heterogeneity were analyzed. This may bias our conclusion for heterogeneity assessment in the study. Sensitivity analysis indicated that after omitting one study after another, the pooled outcomes were robust in this meta-analysis. Despite the existence of heterogeneity and publication bias, the results of this analysis may provide hints and assistances for the field of further research and clinical decision in the diagnosis of CAD. As for research purpose, quantitative assessments and new radiotracers with a lower dose of nuclear exposure to patients and higher spatial resolution need to be further investigated in the context of the results of this meta-analysis. Besides, results of the International Study of Comparative Health Effectiveness With Medical and Invasive Approaches (ISCHEMIA) revealed that, as in comparison with the control group (medical therapy only), the intervention group (angiography, revascularization, and medical therapy) did not reduce the risk of ischemic cardiovascular events or death from any cause over a follow-up of 3.2 years (46, 47). Nevertheless, considering the significant limitations of the study, non-invasive testing will continue to be important to diagnose the etiology of chest pain syndromes, provide prognostic information, guide management decisions, and assess the effectiveness of therapy. In terms of clinical application, physicians' decisions should be made on the basis of level of expertise and the attainability of infrastructure in different clinical settings. Moreover, joint utilization of different detection techniques is recommended to improve diagnostic accuracy.

## Data Availability Statement

The data analyzed in this study is subject to the following licenses/restrictions: The datasets during and/or analyzed during the current study available from the corresponding author on reasonable request. Requests to access these datasets should be directed to Xiaobin Tang, tangxiaobin@nuaa.edu.cn.

## Author Contributions

JX and FC conceived and designed this study. JX and CG were responsible for the collection, extraction, and analysis of the data. JX was responsible for writing the paper. ZW and XT performed the quality evaluation and completed data analysis. XT polished the English language. All authors and participants reviewed the paper and reached an agreement to approve the final manuscript.

## Conflict of Interest

JX and ZW were employed by the company JYAMS PET Research and Development Limited. The remaining authors declare that the research was conducted in the absence of any commercial or financial relationships that could be construed as a potential conflict of interest.

## Abbreviations

CAD: coronary artery disease
ARBs: angiotensin receptor blockers
ACE: angiotensin-converting enzyme
FFR: fractional flow reserve
MPI: myocardial perfusion imaging
CMR: cardiac magnetic resonance
SPECT: single-photon emission computed tomography
PET: positron emission tomography
CT: computed tomography
PRISMA: preferred reporting items for systematic reviews and meta-analysis
TP: true positive
FP: false positive
TN: true negative
FN: false negative
+LR: positive likelihood ratio
–LR: negative likelihood ratio
DOR: diagnostic odds ratio
CI: confidence interval
SROC: summary receiver operating characteristic
AUC: area under the SROC curve
MBF: myocardial blood flow
QUADAS-2: Quality Assessment of Diagnostic Accuracy Studies-2
ISCHEMIA: International Study of Comparative Health Effectiveness With Medical and Invasive Approaches.
