# Supplementary material for: Diagnostic Performance of CMR, SPECT, and PET Imaging for the Identification of Coronary Artery Disease: A Meta-Analysis
Source: Front Cardiovasc Med. 2021 May 7;8:621389. doi: 10.3389/fcvm.2021.621389 (PMC8138058; doi:10.3389/fcvm.2021.621389)
Supplement: Supplementary file 1 [file Data_Sheet_1.pdf]

Diagnostic Performance of CMR, SPECT, and PET Imaging for the Identification of Coronary Artery Disease: A Meta-Analysis

Jianfeng Xu, Fei Cai, Changran Geng, Zheng Wang, Xiaobin Tang

Supplementary Tables and References

Supplementary Table 1. Characteristics of the included CMR studies

Author (Ref.)	Year	No. of Patients	Male,No. (%)	Mean Age, y (SD)	Type of study	Data assessment	Prevalence of CAD (%)	Multivessel disease (%)	Prior myocardial infarction (MI) (%)	Hypertension (%)	Diabetes mellitus (%)	Patient selection
Al-Saadi (1)	2000	40	32 (80)	59 (11)	Prospective	Semiquantitative	100	NS	NS	NS	NS	Suspected CAD
Al-Saadi (2)	2002	23	16 (70)	59 (8)	Prospective	Semiquantitative	100	NS	NS	NS	NS	Known CAD
Bettencourt (3)	2013	103	68 (66)	62 (0.8)	Prospective	Semiquantitative	42	19	NS	73	39	Suspected CAD
Bunce (4)	2004	35	27 (77)	56 (NS)	Prospective	Semiquantitative	49	37	NS	34	9	Suspected CAD
Burgstahler (5)	2008	23	15 (65)	68 (12)	Prospective	Qualitative	40	NS	NS	NS	NS	Suspected CAD
Cheng (6)	2007	65	46 (75)	64 (8)	Prospective	Qualitative	66	32	15	57	16	Suspected CAD
Chiu (7)	2003	13	7 (54)	68	Prospective	Qualitative	92	NS	15	NS	NS	Suspected

				(NS)								CAD
Chirbiri (8)	2013	67	53 (79)	61 (9)	Prospective	Quantitative	82	33	NS	NS	25	Suspected or known CAD
Costa (9)	2007	30	16 (53)	65 (11)	Prospective	Semiquantitative	97	63	NS	80	23	Suspected CAD
Cury (10)	2006	47	38 (81)	63 (5)	Prospective	Qualitative	65	NS	NS	NS	NS	Suspected or known CAD
Doyle (11)	2003	229	0 (0)	59 (11)	Prospective	Semiquantitative	14	2	NS	67	26	Suspected CAD
Ebersberger (12)	2013	116	71 (61)	63 (14)	Prospective	Semiquantitative	78	38	NS	60	26	Suspected or known CAD
Futamatsu (13)	2007	37	16 (53)	65 (11)	Prospective	Semiquantitative	NS	NS	NS	80	23	Suspected CAD
Gebker (14)	2007	40	28 (70)	61 (8)	Prospective	Qualitative	55	40	NS	88	20	Suspected or known CAD
Gebker (15)	2008	414	297 (72)	63 (9)	Prospective	Qualitative	76	29	48	70	28	Suspected or known CAD
Giang (16)	2004	27	24 (89)	58 (10)	Prospective	Semiquantitative	71	33	37	41	19	Suspected CAD
Greenwood (17)	2007	35	31 (89)	55 (9)	Prospective	Qualitative	83	NS	NS	20	17	Suspected CAD
Ghekiere (18)	2019	46	33 (72)	61 (9)	Retrospective	Semiquantitative	33	NS	0	72	26	Suspected

												CAD
Groothuis (19)	2013	192	96 (49)	56 (10)	Prospective	Semiquantitative	55	NS	NS	38	12	Suspected CAD
Hamada (20)	2017	416	297 (71)	63 (11)	Prospective	Semiquantitative	56	NS	NS	76	22	Suspected or known CAD
Ishida (21)	2003	104	81 (78)	66 (12)	Prospective	Qualitative	74	36	NS	NS	NS	Suspected CAD
Jogiya (22)	2012	53	41 (77)	64 (11)	Prospective	Semiquantitative	64	19	NS	66	30	Known or suspected CAD
Kamiya (23)	2014	25	14 (56)	68 (7)	Prospective	Semiquantitative	58	NS	NS	64	60	Known or suspected CAD
Kawase (24)	2004	50	29 (58)	67 (12)	Prospective	Qualitative	66	NS	NS	NS	NS	Suspected CAD
Kitagawa (25)	2008	50	36 (72)	65 (9)	Prospective	Qualitative	72	66	NS	52	46	Suspected or known CAD
Klein (26)	2008	54	35 (65)	60 (10)	Prospective	Qualitative	47	NS	NS	69	22	Suspected CAD
Klem (27)	2006	95	45 (49)	58 (12)	Prospective	Qualitative	48	NS	NS	64	23	Suspected CAD
Klem (28)	2008	147	0 (0)	63 (11)	Prospective	Qualitative	27	NS	NS	68	22	Suspected CAD
Klumpp (29)	2009	57	47 (82)	62 (11)	Prospective	Semiquantitative	72	44	32	68	25	Suspected or known

												CAD
Krittayaphong (30)	2009	66	38 (58)	61 (12)	Prospective	Qualitative	58	74	NS	62	27	Suspected CAD
Kühl (31)	2007	20	13 (68)	64 (13)	Prospective	Semiquantitative	100	41	NS	84	37	Known CAD
Lockie (32)	2011	126	33 (80)	57 (10)	Prospective	Semiquantitative	42	NS	NS	NS	19	Suspected or known CAD
Luu (33)	2014	37	31 (78)	61 (9)	Prospective	Semiquantitative	46	NS	NS	53	25	Suspected or known CAD
Manka (34)	2012	120	90 (75)	64 (12)	Prospective	Semiquantitative	58	13	NS	73	26	Known or suspected CAD
Manka (35)	2015	150	105 (70)	63 (10)	Prospective	Qualitative	65	NS	NS	73	18	Suspected CAD
Merkle (36)	2010	256	179 (70)	62 (NS)	Prospective	Qualitative	72	41	NS	71	7	Suspected CAD
Meyer (37)	2008	60	38 (63)	59 (10)	Prospective	Qualitative	60	53	23	65	23	Suspected CAD
Nagel (38)	2003	90	73 (81)	63 (8)	Prospective	Semiquantitative	51	29	0	0	0	Suspected CAD
Nakamori (39)	2018	96	68 (71)	70 (9)	Prospective	Semiquantitative	96	74	21	71	38	Known or suspected CAD
Nissen (40)	2018	148	88 (59)	62 (8)	Prospective	Semiquantitative	100	47	NS	51	10	Known CAD

Okuda (41)	2005	33	29 (88)	60 (NS)	Prospective	Qualitative	97	NS	NS	NS	NS	Suspected CAD
Papanastasiou (42)	2016	24	20 (83)	63 (7)	Prospective	Semiquantitative	81	NS	NS	54	13	Known or suspected CAD
Paetsch (43)	2004	79	52 (66)	61 (9)	Prospective	Qualitative	67	87	NS	78	24	Suspected or known CAD
Pilz (44)	2006	171	64 (37)	62 (12)	Prospective	Qualitative	66	NS	28	61	27	Suspected or known CAD
Pilz (45)	2008	73	14 (64)	66 (13)	Prospective	Qualitative	30	NS	NS	59	32	Suspected CAD
Pereira (46)	2013	80	54 (68)	61 (8)	Prospective	Qualitative	46	24	NS	72	44	Suspected CAD
Plein (47)	2004	68	54 (79)	57 (11)	Prospective	Qualitative	82	38	13	31	9	Suspected CAD
Plein (48)	2005	92	68 (74)	58 (NS)	Prospective	Semiquantitative	72	NS	21	33	9	Suspected CAD
Ponte (49)	2014	95	65 (68)	62 (8)	Prospective	Qualitative	43	18	NS	75	39	Suspected CAD
Sakuma (50)	2005	40	28 (70)	65 (9)	Prospective	Qualitative	53	28	0	NS	NS	Suspected CAD
SeNSky (51)	2002	30	27 (90)	62 (NS)	Prospective	Qualitative	100	83	70	NS	NS	Known CAD
Takase (52)	2004	102	83 (81)	66 (9)	Prospective	Qualitative	75	NS	44	56	62	Suspected CAD

Thiele (53)	2004	20	21 (66)	64 (8)	Prospective	Semiquantitative	90	28	25	66	34	Suspected CAD
Thomas (54)	2008	60	41 (68)	60 (NS)	Prospective	Qualitative	47	55	47	73	23	Suspected or known CAD
WatkiNS (55)	2009	101	75 (74)	60 (9)	Prospective	Semiquantitative	58	16	0	62	16	Suspected CAD
Wolff (56)	2004	99	62 (83)	57 (NS)	Prospective	Qualitative	54	8	0	NS	NS	Suspected or known CAD

CAD, coronary artery disease. NS, not specified. Ref., reference number. SD, standard deviation.

Supplementary Table 2. Characteristics of SPECT studies included

Author (Ref.)	Year	No. of Patients	Male, No. (%)	Mean Age, y (SD)	Radiotracer	Type of study	Data assessment	Prevalence of CAD (%)	Multivessel disease (%)	Prior myocardial infarction (MI) (%)	Hypertension (%)	Diabetes mellitus (%)	Patient selection
Arbab-Zadeh (57)	2015	391	258 (66)	62 (NS)	99mTc-Tetrofosmin	Prospective	Semiquantitative	60	NS	27	NS	34	Suspected CAD
Aggeli (58)	2007	50	34 (68)	67 (5)	Tl-201	Prospective	Semiquantitative	63	44	0	100	22	Suspected CAD
Amanullah (59)	1997	222	119 (54)	71 (11)	Rest Tl-201 / Stress 99mTc MIBI	Prospective	Semiquantitative	77	41	0	63	29	Suspected CAD
Astarita (60)	2001	53	29 (55)	58 (10)	Tl-201	Prospective	Semiquantitative	43	11	0	100	0	Suspected CAD
Avakian (61)	2001	51	30 (59)	62 (13)	Tl-201	Prospective	Qualitative	31	12	0	NS	NS	Suspected CAD
Baer (62)	1994	35	28 (80)	58 (10)	99mTc- MIBI	Prospective	Qualitative	NS	54	NS	NS	NS	Known CAD
Banzo (63)	2003	99	71 (72)	59 (NS)	99mTc-Tetrofosmin	Prospective	Semiquantitative	52	30	13	34	17	Suspected CAD
Benkiran (64)	2015	70	45 (64)	61 (11)	99mTc-tetrofosmin	Prospective	Quantitative	19	NS	NS	44	36	Suspected or known CAD
Benoit (65)	1996	72	59 (82)	58 (NS)	99mTc-Tetrofosmin	Prospective	Semiquantitative	88	13	57	NS	NS	Suspected or known CAD
Berman (66)	1993	63	46	NS	Rest Tl-201 /	Prospective	Semiquantitative	87	2	NS	NS	NS	Suspected

			(73)		Stress 99mTc MIBI								CAD
Berman (67)	2006	1511	1004 (67)	63 (12)	Rest Tl-201 / Stress 99mTc MIBI	Retrospective	Semiquantitative	77	NS	NS	51	12	Suspected CAD
Budoff (68)	1998	33	19 (58)	55 (9)	99mTc- MIBI	Prospective	Semiquantitative	48	30	0	NS	NS	Suspected CAD
Burns (69)	1991	16	11 (69)	63 (8)	Tl-201	Prospective	Semiquantitative	50	13	6	NS	NS	Suspected or known CAD
Burns (70)	1995	108	48 (86)	59 (9)	Tl-201	Prospective	Qualitative	89	23	NS	NS	NS	Suspected or known CAD
Caobelli (71)	2016	44	31 (70)	65 (11)	99mTc-MIBI	Prospective	Semiquantitative	63	55	45	56	36	Suspected CAD
Cury (72)	2015	110	85 (77)	62 (9)	99mTc-MIBI/99mTc-tetrofosmin	Prospective	Semiquantitative	18	NS	20	70	NS	Suspected or known CAD
Candell-Riera (73)	1997	91	63 (69)	57 (9)	99mTc- MIBI	Prospective	Qualitative	63	52	0	NS	NS	Suspected CAD
Caner (74)	1997	29	15 (52)	NS	Tl-201 (n=27) / 99mTc- MIBI	Prospective	Semiquantitative	NS	NS	0	NS	NS	Suspected or known CAD
Christian (75)	1992	688	528 (77)	63 (NS)	Tl-201	Prospective	Semiquantitative	84	33	42	57	27	Suspected or known CAD
Cramer (76)	1994	76	47 (62)	58 (9)	99mTc- MIBI	Prospective	Qualitative	87	NS	NS	NS	NS	Suspected CAD
Danias (77)	2004	164	105 (64)	62 (11)	99mTc- MIBI	Prospective	Semiquantitative	58	NS	NS	NS	NS	Suspected CAD

Danad (78)	2017	208	132 (64)	58 (9)	99mTc-tetrofosmin	Prospective	Semiquantitative	44	NS	NS	46	16	Suspected CAD
Driessen (79)	2019	157	99 (63)	59 (9)	99mTc-tetrofosmin	Prospective	Quantitative	45	NS	NS	NS	NS	Suspected CAD
Di Bello (80)	1996	45	33 (74)	53 (NS)	99mTc- MIBI	Prospective	Semiquantitative	84	42	16	NS	NS	Suspected CAD
Doyle (11)	2003	184	0 (0)	59 (11)	Tl-201 (n=64) / 99mTc- MIBI (n=165)	Prospective	Qualitative	14	2	NS	67	26	Suspected CAD
Elhendy (81)	2000	124	88 (71)	57 (12)	99mTc- MIBI (n=233) / Tetrofosmin (n=99)	Prospective	Semiquantitative	71	NS	NS	NS	NS	Suspected CAD
Elhendy (82)	2000	135	115 (86)	57 (10)	99mTc-Tetrofosmin	Prospective	Semiquantitative	84	55	100	30	8	Known CAD
Elhendy (83)	2001	332	257 (77)	57 (10)	99mTc- MIBI	Prospective	Semiquantitative	74	33	NS	41	NS	Suspected CAD
Ficaro (84)	1996	60	38 (63)	63 (12)	99mTc- MIBI	Prospective	Semiquantitative	82	8	NS	NS	NS	Suspected or known CAD
Fleming (85)	1991	56	18 (60)	55 (11)	99mTc-Teboroxime	Prospective	Qualitative	68	13	NS	NS	NS	Suspected CAD
Fragasso (86)	1999	101	55 (55)	61 (10)	99mTc- MIBI	Prospective	Semiquantitative	56	37	NS	100	NS	Suspected CAD
Fiechter (87)	2011	66	52 (79)	63 (11)	99mTc-tetrofosmin	Prospective	Qualitative	82	NS	27	83	36	Suspected CAD

Gallowitsch (88)	1998	107	69 (65)	64 (10)	Tl-201	Prospective	Semiquantitative	50	48	54	NS	NS	Suspected CAD
Gentile (89)	2001	132	90 (68)	71 (NS)	Tl-201	Prospective	Qualitative	82	53	NS	NS	NS	Suspected CAD
Genovesi (90)	2011	104	82 (79)	64 (10)	99mTc-tetrofosmin	Prospective	Semiquantitative	61	33	54	77	77	Suspected or known CAD
Go (91)	1990	209	NS	NS	Tl-201	Prospective	Quantitative	75	49	45	NS	NS	Suspected CAD
Groutars (92)	2003	123	89 (72)	61 (10)	Rest Tl-201 / Stress 99mTc MIBI	Prospective	Semiquantitative	88	58	46	33	9	Suspected CAD
Günalp (93)	1993	27	23 (85)	47 (8)	99mTc-MIBI	Prospective	Semiquantitative	67	33	NS	NS	NS	Suspected CAD
Hacot (94)	1993	400	62 (89)	56 (7)	Tl-201	Retrospective	Quantitative	81	46	NS	NS	NS	Suspected or known CAD
Hambÿe (95)	1996	128	90 (70)	60 (9)	99mTc- MIBI	Prospective	Semiquantitative	71	36	0	20	NS	Suspected or known CAD
Hays (96)	1993	144	72 (50)	65 (10)	Tl-201	Prospective	Qualitative	85	31	9	NS	NS	Suspected or known CAD
He (97)	1997	64	41 (64)	57 (NS)	99mTc-Tetrofosmin	Prospective	Qualitative	81	36	0	NS	NS	Suspected or known CAD
Hecht (98)	1993	71	61 (86)	58 (12)	Tl-201	Prospective	Qualitative	72	41	NS	NS	NS	Suspected CAD
Heiba (99)	1997	72	46 (64)	50 (NS)	99mTc- MIBI	Prospective	Semiquantitative	88	33	31	39	31	Suspected or known CAD

Hendel (100)	1999	184	102 (55)	56 (NS)	99mTc- MIBI / Rest Tl-201, Stress 99mTc MIBI	Prospective	Semiquantitative	86	38	15	51	14	Suspected CAD
Ho (101)	1995	54	46 (85)	58 (NS)	Tl-201	Prospective	Qualitative	80	67	41	NS	NS	Suspected or known CAD
Ho (102)	1997	55	39 (77)	56 (NS)	Tl-201	Prospective	Qualitative	75	NS	NS	NS	NS	Suspected CAD
Ho (103)	1998	44	0 (0)	62 (11)	Tl-201	Prospective	Qualitative	55	45	NS	53	25	Suspected or known CAD
Hoffmann (104)	1993	66	51 (77)	57 (10)	99mTc- MIBI	Prospective	Qualitative	76	20	NS	NS	NS	Suspected CAD
Huang (105)	1997	93	72 (77)	61 (10)	Tl-201	Prospective	Qualitative	72	53	39	NS	NS	Suspected or known CAD
Huang (106)	1998	110	81 (74)	61 (NS)	Tl-201	Prospective	Qualitative	59	26	0	NS	NS	Suspected CAD
Iftikhar (107)	1996	92	52 (57)	55 (12)	99mTc- MIBI	Retrospective	Qualitative	74	78	NS	NS	NS	Suspected CAD
Ishida (21)	2003	104	81 (78)	66 (12)	Tl-201 (n=49) / 99mTc- MIBI / Tetrofosmin (n=20)	Prospective	Qualitative	74	36	0	NS	NS	Suspected CAD
Iskandrian (108)	1991	148	85 (57)	63 (9)	Tl-201	Prospective	Qualitative	89	50	25	43	18	Suspected CAD
Ito (109)	2017	72	58 (81)	72 (9)	99mTc- tetrofosmin	Prospective	Semiquantitative	NS	NS	31	76	39	Known or suspected CAD

Jeetley (110)	2006	123	87 (71)	62 (12)	99mTc- MIBI	Prospective	Semiquantitative	78	46	33	59	27	Suspected or known CAD
Kamiya (23)	2014	25	14 (56)	68 (7)	99mTc- tetrofosmin	Prospective	Semiquantitative	80	56	0	64	60	Suspected or known CAD
Kang (111)	1999	326	187 (57)	68 (NS)	Rest Tl-201 / Stress 99mTc MIBI	Retrospective	Semiquantitative	78	49	0	55	42	Suspected CAD
Kapur (112)	2002	2560	1460 (57)	62 (13)	Tl-201 / 99mTc- MIBI / Tetrofosmin	Prospective	Quantitative	69	NS	32	NS	NS	Suspected or known CAD
Karavidas (113)	2006	47	29 (62)	55 (6)	Tl-201	Prospective	Qualitative	23	6	NS	37	12	Non-cardiac perioperative risk stratification
Katayama (114)	2008	46	34 (74)	71 (8)	Tl-201	Prospective	Qualitative	48	24	0	74	33	Suspected or known CAD
Kawai (115)	2004	150	98 (65)	61 (11)	99mTc- Tetrofosmin	Prospective	Qualitative	37	1	NS	65	30	Suspected CAD
Ker (116)	2019	35	17 (49)	53 (9)	99mTc-MIBI	Prospective	Semiquantitative	43	NS	26	NS	40	Suspected CAD
Kiat (117)	1990	53	39 (74)	56 (NS)	99mTc- MIBI	Prospective	Quantitative	91	66	NS	NS	NS	Suspected or known CAD
Kisacik (118)	1996	69	58 (84)	51 (10)	99mTc- MIBI	Prospective	Semiquantitative	68	NS	30	0	NS	Suspected or known CAD
Korosoglou (119)	2006	120	69 (61)	64 (9)	99mTc- MIBI	Prospective	Semiquantitative	70	49	25	59	28	Suspected or known CAD

Kupari (120)	1992	48	23 (52)	63 (NS)	Tl-201	Prospective	Qualitative	48	25	2	NS	NS	Underwent QCA pre-AVR
Li (121)	2012	504	326 (65)	57 (9)	99mTc-MIBI	Prospective	Semiquantitative	40	NS	0	17	6	Suspected CAD
Lipiec (122)	2008	103	65 (63)	58 (9)	99mTc- MIBI	Prospective	Semiquantitative	86	60	62	NS	NS	Suspected or known CAD
Liu (123)	1998	90	68 (76)	59 (7)	Tl-201	Prospective	Qualitative	62	NS	0	61	27	Suspected CAD
Mahmarian (124)	1990	356	265 (74)	56 (10)	Tl-201	Prospective	Quantitative	75	36	NS	NS	NS	Suspected or known CAD
Mairesse (125)	1994	129	95 (74)	NS	99mTc- MIBI	Prospective	Qualitative	64	34	NS	NS	NS	Suspected CAD
Mak (126)	1995	139	114 (82)	51 (10)	99mTc- MIBI	Prospective	Qualitative	87	44	65	55	25	Suspected or known CAD
Marwick (127)	1993	217	156 (72)	58 (10)	99mTc- MIBI	Prospective	Qualitative	65	56	NS	0	NS	Suspected CAD
Marwick (128)	1994	86	60 (70)	59 (9)	99mTc- MIBI	Prospective	Semiquantitative	65	40	0	0	NS	Suspected CAD
Matsumoto (129)	2006	89	82 (92)	62 (11)	99mTc- MIBI	Prospective	Semiquantitative	46	NS	NS	NS	NS	Suspected or known CAD
Matzer (130)	1994	51	25 (49)	67 (11)	Rest Tl-201 / Stress 99mTc MIBI	Prospective	Qualitative	75	49	0	NS	NS	Suspected CAD
McClellan (131)	1996	492	322 (65)	59 (NS)	Tl-201	Prospective	Qualitative	88	26	24	NS	NS	Suspected or known CAD

Meyer (132)	2012	50	37 (74)	61 (10)	99mTc-tetrofosmin	Prospective	Qualitative	60	38	23	65	23	Suspected CAD
Miller (133)	1997	244	241 (99)	63 (9)	99mTc- MIBI	Prospective	Semiquantitative	84	79	32	52	17	Suspected or known CAD
Miller (134)	2002	1853	1376 (74)	63 (11)	Tl-201 (n=10732) / 99mTc- MIBI (3541)	Prospective	Qualitative	72	47	0	53	22	Suspected CAD
Minoves (135)	1993	64	NS	57 (9)	Tl-201 (n=22) / 99mTc- MIBI (n=32)	Prospective	Semiquantitative	56	NS	42	NS	NS	Suspected or known CAD
Nallamothu (136)	1995	321	241 (75)	57 (10)	Tl-201	Prospective	Qualitative	79	50	NS	41	12	Suspected CAD
Neglia (137)	2015	475	291 (61)	60 (9)	NS	Prospective	Qualitative	29	7	0	61	24	Suspected CAD
Nishida (138)	2005	83	55 (66)	68 (NS)	Tl-201	Prospective	Qualitative	NS	53	NS	63	27	Suspected CAD
Nishimura (139)	1991	101	60 (59)	64 (12)	Tl-201	Prospective	Quantitative	69	38	37	NS	NS	Suspected or known CAD
Nguyen (140)	1990	60	39 (65)	62 (8)	Tl-201	Prospective	Qualitative	88	55	37	33	15	Suspected CAD
Ogilby (141)	1992	47	29 (64)	62 (10)	Tl-201	Prospective	Semiquantitative	73	40	20	42	7	Suspected CAD
Oguzhan (142)	1997	70	59 (84)	51 (10)	99mTc- MIBI	Prospective	Semiquantitative	70	46	NS	NS	NS	Suspected or known CAD

Palmas (143)	1995	70	57 (81)	60 (12)	99mTc- MIBI	Prospective	Qualitative	94	51	30	53	11	Suspected or known CAD
Pavlovic (144)	2010	47	16 (37)	55 (9)	99mTc- MIBI	Prospective	Semiquantitative	42	NS	NS	NS	NS	Suspected CAD
Peltier (145)	2004	35	25 (71)	62 (10)	99mTc- MIBI	Prospective	Semiquantitative	63	26	0	66	17	Suspected CAD
Pennell (146)	1991	50	42 (84)	54 (NS)	Tl-201	Prospective	Qualitative	80	52	30	NS	NS	Suspected or known CAD
Pennell (147)	1995	407	298 (73)	58 (NS)	Tl-201	Prospective	Qualitative	80	NS	36	NS	NS	Suspected CAD
Plachcinska (148)	2016	107	65 (61)	62 (8)	99mTc-MIBI	Retrospective	Semiquantitative	39	13	0	79	30	Known or suspected CAD
Pozzoli (149)	1991	75	65 (87)	52 (12)	99mTc- MIBI	Prospective	Semiquantitative	65	21	19	NS	NS	Suspected CAD
Quiñones (150)	1992	292	195 (67)	57 (8)	Tl-201	Prospective	Qualitative	77	15	NS	NS	NS	Suspected or known CAD
Sakuma (50)	2005	40	28 (70)	65 (9)	Tl-201	Prospective	Qualitative	53	28	0	NS	NS	Suspected CAD
Salustri (151)	1992	44	35 (80)	59 (9)	Tl-201 (n=19) / 99mTc- MIBI (n=25)	Prospective	Semiquantitative	68	0	36	NS	NS	Suspected or known CAD
San Román (152)	1998	102	50 (49)	64 (10)	99mTc- MIBI	Prospective	Semiquantitative	67	33	NS	NS	NS	Suspected CAD
Sandler (153)	1995	65	14 (61)	54 (16)	99mTc- MIBI	Prospective	Qualitative	61	NS	NS	NS	NS	Suspected or known CAD

Santoro (154)	1998	60	NS	NS	99mTc- MIBI	Prospective	Qualitative	55	NS	NS	NS	NS	Suspected CAD
Sand (155)	2018	143	84 (59)	64 (11)	99mTc- tetrofosmin	Prospective	Qualitative	63	17	NS	62	12	Suspected CAD
Schaap (156)	2013	98	67 (68)	63 (10)	99mTc-MIBI	Prospective	Semiquantitative	57	42	NS	63	12	Suspected CAD
Schwartz (157)	2003	149	98 (73)	63 (14)	Rest Tl-201 / Stress 99mTc MIBI	Prospective	Qualitative	NS	NS	24	NS	NS	Suspected or known CAD
Senior (158)	2004	55	45 (82)	NS	99mTc- Tetrofosmin	Prospective	Qualitative	78	20	NS	40	9	Suspected CAD
Sharir (159)	2000	99	80 (81)	67 (10)	Rest Tl-201 / Stress 99mTc MIBI	Prospective	Semiquantitative	NS	61	NS	NS	NS	Suspected CAD
Smart (160)	2000	183	133 (73)	60 (11)	99mTc- MIBI	Prospective	Semiquantitative	65	32	NS	NS	NS	Suspected or known CAD
Solot (161)	1993	128	79 (62)	61 (NS)	99mTc- MIBI	Prospective	Qualitative	70	NS	NS	NS	NS	Suspected CAD
Soman (162)	1997	27	18 (67)	58 (7)	99mTc- MIBI	Prospective	Qualitative	78	66	NS	NS	NS	Suspected CAD
Squires (163)	2005	51	24 (47)	60 (NS)	99mTc- Tetrofosmin	Prospective	Qualitative	62	NS	NS	69	37	Suspected CAD
Stewart (164)	1991	81	52 (64)	57 (12)	Tl-201	Retrospective	Qualitative	74	NS	47	NS	NS	Suspected CAD
Sylvén (165)	1994	160	100	58	99mTc- MIBI	Prospective	Semiquantitative	85	NS	NS	NS	NS	Suspected

			(63)	(NS)									CAD
Tadehara (166)	2008	422	219 (52)	72 (10)	99mTc- MIBI	Prospective	Qualitative	53	NS	NS	NS	NS	Suspected or known CAD
Taillefer (167)	1997	85	0 (0)	60 (NS)	99mTc- MIBI	Prospective	Semiquantitative	75	NS	14	NS	NS	Suspected or known CAD
Takeishi (168)	1994	42	29 (69)	68 (NS)	99mTc- MIBI	Prospective	Semiquantitative	81	NS	NS	NS	NS	Suspected CAD
Takeuchi (169)	1993	120	89 (74)	63 (NS)	Tl-201	Prospective	Qualitative	62	31	NS	NS	NS	Suspected or known CAD
Tartagni (170)	1991	30	26 (87)	59 (8)	99mTc- MIBI	Prospective	Semiquantitative	87	60	57	NS	NS	Suspected or known CAD
Thiele (53)	2004	32	21 (66)	64 (8)	99mTc-Tetrofosmin	Prospective	Qualitative	NS	28	NS	66	34	Suspected or known CAD
Travin (171)	2000	107	49 (46)	68 (NS)	99mTc- MIBI	Prospective	Semiquantitative	NS	39	41	69	38	Suspected or known CAD
Tsai (172)	2002	240	199 (83)	56 (9)	Tl-201	Prospective	Qualitative	73	49	41	47	16	Suspected or known CAD
Tsutsui (173)	2005	36	15 (42)	62 (14)	Rest Tl-201 / Stress 99mTc MIBI	Retrospective	Qualitative	NS	19	0	86	47	Suspected or known CAD
van Diemen (174)	2020	169	101 (60)	58 (9)	99mTc-tetrofosmin	Prospective	Qualitative	52	NS	NS	49	17	Suspected CAD
Van Train (175)	1990	371	319 (86)	NS	99mTc- MIBI	Prospective	Qualitative	83	59	35	NS	NS	Suspected or known CAD
Van Train	1993	40	33	60	99mTc- MIBI	Prospective	Quantitative	76	33	NS	NS	NS	Suspected or

(176)			(83)	(NS)									known CAD
Van Train (177)	1994	161	122 (76)	56 (NS)	Tl-201	Prospective	Quantitative	82	22	0	NS	NS	Suspected or known CAD
Verani (178)	1990	89	44 (49)	64 (10)	Tl-201	Prospective	Quantitative	64	NS	35	NS	NS	Suspected CAD / preoperative risk stratification
Wang (179)	1995	75	39 (52)	84 (NS)	99mTc- MIBI	Prospective	Qualitative	84	45	0	52	16	Suspected CAD
Watanabe (180)	1997	70	43 (61)	62 (10)	Tl-201	Prospective	Semiquantitative	66	29	19	30	20	Suspected or known
Weinsaft (181)	2007	183	95 (52)	NS	Rest Tl-201 / Stress 99mTc MIBI	Prospective	Qualitative	47	31	NS	NS	20	Suspected or known CAD
Wu (182)	2009	218	135 (62)	64 (11)	99mTc- MIBI	Prospective	Semiquantitative	60	37	NS	70	40	Suspected or known CAD
Xin (183)	2019	181	123 (68)	62 (9)	99mTc-MIBI	Retrospective	Quantitative	41	51	0	71	22	Suspected CAD
Yeih (184)	2007	51	0 (0)	63 (9)	Tl-201	Prospective	Qualitative	55	61	14	71	26	Suspected CAD

CAD, coronary artery disease. NS, not specified. Ref., reference number. SD, standard deviation.

Supplementary Table 3. Characteristics of the included PET studies

Author (Ref.)	Year	No. of Patients	Male, No. (%)	Mean Age, y (SD)	PET Radiotracer	Type of study	Data assessment	Prevalence of CAD (%)	Multivessel disease (%)	Prior myocardial infarction (MI) (%)	Hypertension (%)	Diabetes mellitus (%)	Patient selection
Aggarwal (185)	2015	265	187 (71)	62 (10)	N-13	Prospective	Semiquantitative	31	5	NS	71	32	Suspected or known CAD
Bateman (186)	2006	112	61 (52)	67 (NS)	Rb-82	Retrospective	Qualitative	82	71	25	77	33	Suspected or known CAD
Botsch (187)	1994	52	NS	56 (NS)	Rb-82	Prospective	Qualitative	74	38	38	NS	NS	Suspected or known CAD
Chow (188)	2007	26	18 (69)	57 (9)	Rb-82	Prospective	Semiquantitative	79	35	15	62	15	Suspected or known CAD
Danad (78)	2017	208	132 (64)	58 (9)	O-15	Prospective	Semiquantitative	44	10	NS	46	16	Suspected CAD
Dekker (189)	2020	150	96 (64)	68 (12)	Rb-82	Retrospective	Semiquantitative	58	NS	12	65	23	Suspected CAD
Driessen (79)	2019	157	99 (63)	59 (9)	O-15	Prospective	Quantitative	45	NS	NS	NS	NS	Suspected CAD
Esteves (190)	2008	75	28 (54)	67 (11)	Rb-82	Retrospective	Qualitative	85	NS	NS	73	31	Suspected CAD
Fathala (191)	2019	383	215 (56)	64 (11)	N-13	Retrospective	Qualitative	NS	NS	NS	91	71	Suspected CAD
Go (91)	1990	209	NS	NS	Rb-82	Prospective	Quantitative	75	49	45	NS	NS	Suspected CAD

Grover-McKay (192)	1992	31	15 (94)	60 (11)	Rb-82	Prospective	Quantitative	100	35	13	NS	NS	Suspected or known CAD
Kaster (193)	2012	70	51 (73)	64 (12)	Rb-82	Prospective	Semiquantitative	59	NS	34	76	43	Suspected or known CAD
Laubenbacher (194)	1993	52	NS	61 (11)	N-13	Prospective	Quantitative	55	13	NS	NS	NS	Suspected CAD
Lee (195)	2016	130	116 (90)	64 (9)	N-13	Prospective	Quantitative	NS	75	0	69	35	Suspected CAD
Marwick (196)	1992	74	60 (81)	60 (4)	Rb-82	Prospective	Qualitative	95	43	49	NS	NS	Known CAD
Neglia (137)	2015	475	291 (61)	60 (9)	NS	Prospective	Qualitative	29	7	0	61	24	Suspected CAD
Sampson (197)	2007	64	39 (61)	62 (15)	Rb-82	Prospective	Qualitative	69	31	0	86	36	Suspected CAD
Santana (198)	2007	53	29 (55)	NS	Rb-82	Prospective	Quantitative	85	57	NS	85	44	Suspected or known CAD
Shi (199)	2007	95	32 (49)	63 (11)	Rb-82	Prospective	Qualitative	88	38	NS	NS	NS	Suspected or known CAD
Simone (200)	1992	225	180 (80)	57 (NS)	Rb-82	Retrospective	Qualitative	NS	NS	NS	NS	NS	Suspected CAD
Stewart (164)	1991	81	52 (64)	57 (12)	Rb-82	Prospective	Semiquantitative	74	NS	47	NS	NS	Suspected CAD
Strähl (201)	2020	176	142 (80)	NS	N-13	Retrospective	Quantitative	NS	NS	NS	76	39	Suspected CAD

van Diemen (174)	2020	169	101 (60)	58 (9)	O-15	Prospective	Qualitative	52	NS	NS	49	17	Suspected CAD
Wallhaus (202)	2001	28	24 (86)	62 (NS)	Cu-62	Prospective	Qualitative	82	61	0	0	NS	Suspected or known CAD
Williams (203)	1994	287	215 (75)	NS	Rb-82	Retrospective	Qualitative	NS	NS	NS	NS	NS	Suspected CAD

CAD, coronary artery disease. NS, not specified. Ref., reference number. SD, standard deviation.

Supplementary Table 4. Subgroup analyses for the diagnostic performance of CMR on patient-based level

Characteristics	Sensitivity	Specificity	+LR	-LR	DOR	SROC Curve AUC
**Type of study**						
Prospective	0.89 [0.85, 0.91]	0.80 [0.76, 0.83]	4.4 [3.7, 5.4]	0.14 [0.11, 0.19]	32 [21, 46]	0.90 [0.87, 0.93]
**Data assessment**						
Qualitative	0.89 [0.87, 0.91]	0.79 [0.74, 0.84]	4.4 [3.4, 5.5]	0.13 [0.11, 0.16]	33 [22, 50]	0.92 [0.90, 0.94]
Semiquantitative	0.85 [0.72, 0.92]	0.80 [0.76, 0.83]	4.2 [3.4, 5.2]	0.19 [0.10, 0.36]	22 [10, 47]	0.81 [0.77, 0.84]
**Prevalence of CAD (%)**						
<60	0.85 [0.81, 0.89]	0.78 [0.71, 0.84]	3.9 [2.9, 5.2]	0.19 [0.15, 0.25]	20 [13, 33]	0.89 [0.86, 0.91]
≥60	0.90 [0.85, 0.94]	0.81 [0.78, 0.84]	4.8 [4.0, 5.8]	0.12 [0.08, 0.18]	40 [24, 66]	0.82[0.79, 0.86]
**Multivessel disease (%)**						
<50	0.87 [0.81, 0.92]	0.81 [0.78, 0.84]	4.6 [3.8, 5.6]	0.16 [0.11, 0.24]	29 [17, 49]	0.86 [0.82, 0.88]
≥50	0.89 [0.84, 0.93]	0.80 [0.72, 0.86]	4.4 [3.1, 6.3]	0.14 [0.09, 0.21]	32 [17, 60]	0.92 [0.89, 0.94]
**Patient selection**						
Suspected CAD	0.88 [0.85, 0.91]	0.80 [0.74, 0.85]	4.4 [3.3, 5.9]	0.15 [0.11, 0.19]	30 [19, 48]	0.92 [0.89, 0.94]
Suspected or known CAD	0.90 [0.86, 0.93]	0.79 [0.74, 0.83]	4.2 [3.4, 5.3]	0.13 [0.09, 0.18]	34 [20, 55]	0.88 [0.85, 0.91]

CMR, cardiac magnetic resonance. CAD, coronary artery disease. +LR, positive likelihood ratio. -LR, negative likelihood ratio. DOR, diagnostic odds ratio. SROC, summary receiver operating characteristic. AUC, area under the SROC curve.

Supplementary Table 5. Subgroup analyses for the diagnostic performance of SPECT on patient-based level

Characteristics	Sensitivity	Specificity	+LR	-LR	DOR	SROC Curve AUC
**Type of study**						
Prospective	0.88 [0.86, 0.90]	0.73 [0.69, 0.77]	3.3 [2.8, 3.8]	0.16 [0.14, 0.19]	20 [16, 25]	0.89 [0.86, 0.91]
Retrospective	0.88 [0.84, 0.90]	0.56 [0.39, 0.72]	2.0 [1.3, 3.0]	0.22 [0.14, 0.36]	9 [4, 22]	0.87 [0.84, 0.90]
**Data assessment**						
Qualitative	0.88 [0.85, 0.91]	0.74 [0.69, 0.79]	3.4 [2.8, 4.2]	0.16 [0.13, 0.20]	21 [16, 30]	0.89 [0.86, 0.92]
Semiquantitative	0.87 [0.84, 0.90]	0.70 [0.63, 0.75]	2.9 [2.4, 3.5]	0.18 [0.15, 0.23]	16 [11, 22]	0.88 [0.85, 0.90]
Quantitative	0.90 [0.86, 0.93]	0.72 [0.58, 0.83]	3.3 [2.1, 5.1]	0.14 [0.10, 0.19]	24 [13, 43]	0.91 [0.88, 0.93]
**Prevalence of CAD (%)**						
<60	0.86 [0.80, 0.91]	0.76 [0.68, 0.82]	3.6 [2.7, 4.8]	0.18 [0.13, 0.26]	20 [12, 32]	0.89 [0.86, 0.91]
≥60	0.88 [0.86, 0.90]	0.71 [0.66, 0.75]	3.1 [2.6, 3.6]	0.16 [0.14, 0.19]	19 [14, 24]	0.89 [0.86, 0.91]
**Multivessel disease (%)**						
<50	0.87 [0.84, 0.89]	0.72 [0.66, 0.76]	3.1 [2.6, 3.6]	0.19 [0.16, 0.22]	16 [13, 21]	0.88 [0.85, 0.91]
≥50	0.91 [0.88, 0.94]	0.72 [0.62, 0.80]	3.3 [2.3, 4.5]	0.12 [0.09, 0.17]	27 [16, 47]	0.92 [0.89, 0.94]
**Patient selection**						
Suspected CAD	0.86 [0.83, 0.89]	0.74 [0.68, 0.79]	3.3 [2.7, 4.0]	0.18 [0.15, 0.23]	18 [13, 25]	0.88 [0.85, 0.91]

Suspected or known CAD	0.89 [0.87, 0.91]	0.71 [0.65, 0.76]	3.1 [2.5, 3.7]	0.15 [0.13, 0.18]	20 [15, 27]	0.90 [0.87, 0.92]

SPECT, single photon emission computed tomography. CAD, coronary artery disease. +LR, positive likelihood ratio. -LR, negative likelihood ratio. DOR, diagnostic odds ratio. SROC, summary receiver operating characteristic. AUC, area under the SROC curve.

Supplementary Table 6. Subgroup analyses for the diagnostic performance of PET on patient-based level

Characteristics	Sensitivity	Specificity	+LR	-LR	DOR	SROC Curve AUC
**Type of study**						
Prospective	0.88 [0.74, 0.95]	0.88 [0.66, 0.96]	7.2 [2.5, 21.0]	0.14 [0.07, 0.28]	53 [21, 130]	0.94 [0.92, 0.96]
**Data assessment**						
Qualitative	0.84 [0.65, 0.94]	0.97 [0.58, 1.00]	32.9 [1.5, 738.1]	0.16 [0.07, 0.37]	206 [14, 3078]	0.95 [0.93, 0.97]
**Prevalence of CAD (%)**						
≥60	0.88 [0.77, 0.94]	0.90 [0.72, 0.97]	9.2 [3.0, 27.9]	0.14 [0.08, 0.24]	68 [27, 171]	0.95 [0.92, 0.96]
**Multivessel disease (%)**						
<50	0.88 [0.66, 0.97]	0.88 [0.50, 0.98]	7.3 [1.4, 37.5]	0.14 [0.05, 0.38]	53 [14, 207]	0.94 [0.92, 0.96]
**Patient selection**						
Suspected CAD	0.91 [0.85, 0.94]	0.83 [0.58, 0.94]	5.3 [1.9, 14.3]	0.11 [0.07, 0.17]	47 [17, 131]	0.94 [0.91, 0.95]
Suspected or known CAD	0.86 [0.61, 0.96]	0.92 [0.71, 0.98]	10.7 [2.9, 40.1]	0.15 [0.05, 0.47]	69 [17, 287]	0.95 [0.93, 0.97]

PET, positron emission tomography. CAD, coronary artery disease. +LR, positive likelihood ratio. -LR, negative likelihood ratio. DOR, diagnostic odds ratio. SROC, summary receiver operating characteristic. AUC, area under the SROC curve.

References

1. Al-Saadi N, Nagel E, Gross M et al. Noninvasive detection of myocardial ischemia from perfusion reserve based on cardiovascular magnetic resonance. Circulation 2000;101:1379-83.
2. Al-Saadi N, Gross M, Paetsch I et al. Dobutamine induced myocardial perfusion reserve index with cardiovascular MR in patients with coronary artery disease. Journal of cardiovascular magnetic resonance : official journal of the Society for Cardiovascular Magnetic Resonance 2002;4:471-80.
3. Bettencourt N, Chiribiri A, Schuster A et al. Cardiac magnetic resonance myocardial perfusion imaging for detection of functionally significant obstructive coronary artery disease: a prospective study. International journal of cardiology 2013;168:765-73.
4. Bunce NH, Reyes E, Keegan J et al. Combined coronary and perfusion cardiovascular magnetic resonance for the assessment of coronary artery stenosis. Journal of cardiovascular magnetic resonance : official journal of the Society for Cardiovascular Magnetic Resonance 2004;6:527-39.
5. Burgstahler C, Kunze M, Gawaz MP et al. Adenosine stress first pass perfusion for the detection of coronary artery disease in patients with aortic stenosis: a feasibility study. The international journal of cardiovascular imaging 2008;24:195-200.
6. Cheng AS, Pegg TJ, Karamitsos TD et al. Cardiovascular magnetic resonance perfusion imaging at 3-tesla for the detection of coronary artery disease: a comparison with 1.5-tesla. Journal of the American College of Cardiology 2007;49:2440-9.
7. Chiu CW, So NM, Lam WW, Chan KY, Sanderson JE. Combined first-pass perfusion and viability study at MR imaging in patients with non-ST segment-elevation acute coronary syndromes: feasibility study. Radiology 2003;226:717-22.
8. Chiribiri A, Hautvast GL, Lockie T et al. Assessment of coronary artery stenosis severity and location: quantitative analysis of transmural perfusion gradients by high-resolution MRI versus FFR. JACC Cardiovascular imaging 2013;6:600-9.
9. Costa MA, Shoemaker S, Futamatsu H et al. Quantitative magnetic resonance perfusion imaging detects anatomic and physiologic coronary artery disease as measured by coronary angiography and fractional flow reserve. Journal of the American College of Cardiology 2007;50:514-22.
10. Cury RC, Cattani CA, Gabure LA et al. Diagnostic performance of stress perfusion and delayed-enhancement MR imaging in patients with coronary artery disease. Radiology 2006;240:39-45.
11. Doyle M, Fuisz A, Kortright E et al. The impact of myocardial flow reserve on the detection of coronary artery disease by perfusion imaging methods: an NHLBI WISE study. Journal of cardiovascular magnetic resonance : official journal of the Society for Cardiovascular Magnetic Resonance 2003;5:475-85.
12. Ebersberger U, Makowski MR, Schoepf UJ et al. Magnetic resonance myocardial perfusion imaging at 3.0 Tesla for the identification of myocardial ischaemia: comparison with coronary catheter angiography and fractional flow reserve measurements. European heart journal cardiovascular Imaging 2013;14:1174-80.

13. Futamatsu H, Wilke N, Klassen C et al. Evaluation of cardiac magnetic resonance imaging parameters to detect anatomically and hemodynamically significant coronary artery disease. American heart journal 2007;154:298-305.
14. Gebker R, Jahnke C, Paetsch I et al. MR myocardial perfusion imaging with k-space and time broad-use linear acquisition speed-up technique: feasibility study. Radiology 2007;245:863-71.
15. Gebker R, Jahnke C, Manka R et al. Additional value of myocardial perfusion imaging during dobutamine stress magnetic resonance for the assessment of coronary artery disease. Circulation Cardiovascular imaging 2008;1:122-30.
16. Giang TH, Nanz D, Coulden R et al. Detection of coronary artery disease by magnetic resonance myocardial perfusion imaging with various contrast medium doses: first European multi-centre experience. European heart journal 2004;25:1657-65.
17. Greenwood JP, Younger JF, Ridgway JP, Sivananthan MU, Ball SG, Plein S. Safety and diagnostic accuracy of stress cardiac magnetic resonance imaging vs exercise tolerance testing early after acute ST elevation myocardial infarction. Heart (British Cardiac Society) 2007;93:1363-8.
18. Ghekiere O, Dacher JN, Dewilde W et al. Value of Relative Myocardial Perfusion at MRI for Fractional Flow Reserve-Defined Ischemia: A Pilot Study. AJR American journal of roentgenology 2019:1-8.
19. Groothuis JG, Beek AM, Brinckman SL et al. Combined non-invasive functional and anatomical diagnostic work-up in clinical practice: the magnetic resonance and computed tomography in suspected coronary artery disease (MARCC) study. European heart journal 2013;34:1990-8.
20. Hamada S, Gotschy A, Wissmann L et al. Multi-centre study of whole-heart dynamic 3D cardiac magnetic resonance perfusion imaging for the detection of coronary artery disease defined by fractional flow reserve: gender based analysis of diagnostic performance. European heart journal cardiovascular Imaging 2017;18:1099-1106.
21. Ishida N, Sakuma H, Motoyasu M et al. Noninfarcted myocardium: correlation between dynamic first-pass contrast-enhanced myocardial MR imaging and quantitative coronary angiography. Radiology 2003;229:209-16.
22. Jogiya R, Kozerke S, Morton G et al. Validation of dynamic 3-dimensional whole heart magnetic resonance myocardial perfusion imaging against fractional flow reserve for the detection of significant coronary artery disease. Journal of the American College of Cardiology 2012;60:756-65.
23. Kamiya K, Sakakibara M, Asakawa N et al. Cardiac magnetic resonance performs better in the detection of functionally significant coronary artery stenosis compared to single-photon emission computed tomography and dobutamine stress echocardiography. Circulation journal : official journal of the Japanese Circulation Society 2014;78:2468-76.
24. Kawase Y, Nishimoto M, Hato K, Okajima K, Yoshikawa J. Assessment of coronary artery disease with nicorandil stress magnetic resonance imaging. Osaka City Med J 2004;50:87-94.
25. Kitagawa K, Sakuma H, Nagata M et al. Diagnostic accuracy of stress myocardial perfusion MRI and late gadolinium-enhanced MRI for detecting flow-limiting coronary artery disease: a multicenter study. European radiology 2008;18:2808-16.

26. Klein C, Gebker R, Kokocinski T et al. Combined magnetic resonance coronary artery imaging, myocardial perfusion and late gadolinium enhancement in patients with suspected coronary artery disease. Journal of cardiovascular magnetic resonance : official journal of the Society for Cardiovascular Magnetic Resonance 2008;10:45.
27. Klem I, Heitner JF, Shah DJ et al. Improved detection of coronary artery disease by stress perfusion cardiovascular magnetic resonance with the use of delayed enhancement infarction imaging. Journal of the American College of Cardiology 2006;47:1630-8.
28. Klem I, Greulich S, Heitner JF et al. Value of cardiovascular magnetic resonance stress perfusion testing for the detection of coronary artery disease in women. JACC Cardiovascular imaging 2008;1:436-45.
29. Klumpp BD, Seeger A, Doesch C et al. High resolution myocardial magnetic resonance stress perfusion imaging at 3 T using a 1 M contrast agent. European radiology 2010;20:533-41.
30. Krittayaphong R, Boonyasirinant T, Saiviroonporn P et al. Myocardial perfusion cardiac magnetic resonance for the diagnosis of coronary artery disease: do we need rest images? The international journal of cardiovascular imaging 2009;25 Suppl 1:139-48.
31. Kuhl HP, Katoh M, Buhr C et al. Comparison of magnetic resonance perfusion imaging versus invasive fractional flow reserve for assessment of the hemodynamic significance of epicardial coronary artery stenosis. Am J Cardiol 2007;99:1090-5.
32. Lockie T, Ishida M, Perera D et al. High-resolution magnetic resonance myocardial perfusion imaging at 3.0-Tesla to detect hemodynamically significant coronary stenoses as determined by fractional flow reserve. Journal of the American College of Cardiology 2011;57:70-5.
33. Luu JM, Friedrich MG, Harker J et al. Relationship of vasodilator-induced changes in myocardial oxygenation with the severity of coronary artery stenosis: a study using oxygenation-sensitive cardiovascular magnetic resonance. European heart journal cardiovascular Imaging 2014;15:1358-67.
34. Manka R, Paetsch I, Kozerke S et al. Whole-heart dynamic three-dimensional magnetic resonance perfusion imaging for the detection of coronary artery disease defined by fractional flow reserve: determination of volumetric myocardial ischaemic burden and coronary lesion location. European heart journal 2012;33:2016-24.
35. Manka R, Wissmann L, Gebker R et al. Multicenter evaluation of dynamic three-dimensional magnetic resonance myocardial perfusion imaging for the detection of coronary artery disease defined by fractional flow reserve. Circulation Cardiovascular imaging 2015;8.
36. Merkle N, Wohrle J, Nusser T et al. Diagnostic performance of magnetic resonance first pass perfusion imaging is equally potent in female compared to male patients with coronary artery disease. Clin Res Cardiol 2010;99:21-8.
37. Meyer C, Strach K, Thomas D et al. High-resolution myocardial stress perfusion at 3 T in patients with suspected coronary artery disease. European radiology 2008;18:226-33.
38. Nagel E, Klein C, Paetsch I et al. Magnetic resonance perfusion measurements for the noninvasive detection of coronary artery disease. Circulation 2003;108:432-7.

39. Nakamori S, Sakuma H, Dohi K et al. Combined Assessment of Stress Myocardial Perfusion Cardiovascular Magnetic Resonance and Flow Measurement in the Coronary Sinus Improves Prediction of Functionally Significant Coronary Stenosis Determined by Fractional Flow Reserve in Multivessel Disease. Journal of the American Heart Association 2018;7.
40. Nissen L, Winther S, Westra J et al. Diagnosing coronary artery disease after a positive coronary computed tomography angiography: the Dan-NICAD open label, parallel, head to head, randomized controlled diagnostic accuracy trial of cardiovascular magnetic resonance and myocardial perfusion scintigraphy. European heart journal cardiovascular Imaging 2018;19:369-377.
41. Okuda S, Tanimoto A, Satoh T et al. Evaluation of ischemic heart disease on a 1.5 Tesla scanner: combined first-pass perfusion and viability study. Radiat Med 2005;23:230-5.
42. Papanastasiou G, Williams MC, Dweck MR et al. Quantitative assessment of myocardial blood flow in coronary artery disease by cardiovascular magnetic resonance: comparison of Fermi and distributed parameter modeling against invasive methods. Journal of cardiovascular magnetic resonance : official journal of the Society for Cardiovascular Magnetic Resonance 2016;18:57.
43. Paetsch I, Jahnke C, Wahl A et al. Comparison of dobutamine stress magnetic resonance, adenosine stress magnetic resonance, and adenosine stress magnetic resonance perfusion. Circulation 2004;110:835-42.
44. Pilz G, Bernhardt P, Klos M, Ali E, Wild M, Hofling B. Clinical implication of adenosine-stress cardiac magnetic resonance imaging as potential gatekeeper prior to invasive examination in patients with AHA/ACC class II indication for coronary angiography. Clin Res Cardiol 2006;95:531-8.
45. Pilz G, Klos M, Ali E, Hoefling B, Scheck R, Bernhardt P. Angiographic correlations of patients with small vessel disease diagnosed by adenosine-stress cardiac magnetic resonance imaging. Journal of cardiovascular magnetic resonance : official journal of the Society for Cardiovascular Magnetic Resonance 2008;10:8.
46. Pereira E, Bettencourt N, Ferreira N et al. Incremental value of adenosine stress cardiac magnetic resonance in coronary artery disease detection. International journal of cardiology 2013;168:4160-7.
47. Plein S, Greenwood JP, Ridgway JP, Cranny G, Ball SG, Sivananthan MU. Assessment of non-ST-segment elevation acute coronary syndromes with cardiac magnetic resonance imaging. Journal of the American College of Cardiology 2004;44:2173-81.
48. Plein S, Radjenovic A, Ridgway JP et al. Coronary artery disease: myocardial perfusion MR imaging with sensitivity encoding versus conventional angiography. Radiology 2005;235:423-30.
49. Ponte M, Bettencourt N, Pereira E et al. Anatomical versus functional assessment of coronary artery disease: direct comparison of computed tomography coronary angiography and magnetic resonance myocardial perfusion imaging in patients with intermediate pre-test probability. The international journal of cardiovascular imaging 2014;30:1589-97.

50. Sakuma H, Suzawa N, Ichikawa Y et al. Diagnostic accuracy of stress first-pass contrast-enhanced myocardial perfusion MRI compared with stress myocardial perfusion scintigraphy. AJR American journal of roentgenology 2005;185:95-102.
51. Sensky PR, Samani NJ, Reek C, Cherryman GR. Magnetic resonance perfusion imaging in patients with coronary artery disease: a qualitative approach. The international journal of cardiovascular imaging 2002;18:373-83; discussion 385-6.
52. Takase B, Nagata M, Kihara T et al. Whole-heart dipyridamole stress first-pass myocardial perfusion MRI for the detection of coronary artery disease. Jpn Heart J 2004;45:475-86.
53. Thiele H, Plein S, Breeuwer M et al. Color-encoded semiautomatic analysis of multi-slice first-pass magnetic resonance perfusion: comparison to tetrofosmin single photon emission computed tomography perfusion and X-ray angiography. The international journal of cardiovascular imaging 2004;20:371-84; discussion 385-7.
54. Thomas D, Strach K, Meyer C et al. Combined myocardial stress perfusion imaging and myocardial stress tagging for detection of coronary artery disease at 3 Tesla. Journal of cardiovascular magnetic resonance : official journal of the Society for Cardiovascular Magnetic Resonance 2008;10:59.
55. Watkins S, McGeoch R, Lyne J et al. Validation of magnetic resonance myocardial perfusion imaging with fractional flow reserve for the detection of significant coronary heart disease. Circulation 2009;120:2207-13.
56. Wolff SD, Schwitter J, Coulden R et al. Myocardial first-pass perfusion magnetic resonance imaging: a multicenter dose-ranging study. Circulation 2004;110:732-7.
57. Arbab-Zadeh A, Di Carli MF, Cerci R et al. Accuracy of Computed Tomographic Angiography and Single-Photon Emission Computed Tomography-Acquired Myocardial Perfusion Imaging for the Diagnosis of Coronary Artery Disease. Circulation Cardiovascular imaging 2015;8:e003533.
58. Aggeli C, Christoforatou E, Giannopoulos G et al. The diagnostic value of adenosine stress-contrast echocardiography for diagnosis of coronary artery disease in hypertensive patients: comparison to Tl-201 single-photon emission computed tomography. Am J Hypertens 2007;20:533-8.
59. Amanullah AM, Berman DS, Kiat H, Friedman JD. Usefulness of hemodynamic changes during adenosine infusion in predicting the diagnostic accuracy of adenosine technetium-99m sestamibi single-photon emission computed tomography (SPECT). The American journal of cardiology 1997;79:1319-22.
60. Astarita C, Palinkas A, Nicolai E, Maresca FS, Varga A, Picano E. Dipyridamole-atropine stress echocardiography versus exercise SPECT scintigraphy for detection of coronary artery disease in hypertensives with positive exercise test. J Hypertens 2001;19:495-502.
61. Avakian SD, Grinberg M, Meneguetti JC, Ramires JA, Mansur AP. SPECT dipyridamole scintigraphy for detecting coronary artery disease in patients with isolated severe aortic stenosis. International journal of cardiology 2001;81:21-7.
62. Baer FM, Voth E, Theissen P, Schneider CA, Schicha H, Sechtem U. Coronary artery disease: findings with GRE MR imaging and Tc-99m-methoxyisobutyl-isonitrile SPECT during simultaneous dobutamine stress. Radiology 1994;193:203-9.

63. Banzo I, Pena FJ, Allende RH, Quirce R, Carril JM. Prospective clinical comparison of non-corrected and attenuation- and scatter-corrected myocardial perfusion SPECT in patients with suspicion of coronary artery disease. Nuclear medicine communications 2003;24:995-1002.
64. Benkiran M, Mariano-Goulart D, Bourdon A, Sibille L, Bouallègue FB. Is computed tomography attenuation correction more efficient than gated single photon emission computed tomography analysis in improving the diagnostic performance of myocardial perfusion imaging in patients with low prevalence of ischemic heart disease? Nuclear medicine communications 2015;36:69-77.
65. Benoit T, Vivegnis D, Lahiri A, Itti R, Braat S, Rigo P. Tomographic myocardial imaging with technetium-99m tetrofosmin. Comparison with tetrofosmin and thallium planar imaging and with angiography. European heart journal 1996;17:635-42.
66. Berman DS, Kiat H, Friedman JD et al. Separate acquisition rest thallium-201/stress technetium-99m sestamibi dual-isotope myocardial perfusion single-photon emission computed tomography: a clinical validation study. Journal of the American College of Cardiology 1993;22:1455-64.
67. Berman DS, Kang X, Nishina H et al. Diagnostic accuracy of gated Tc-99m sestamibi stress myocardial perfusion SPECT with combined supine and prone acquisitions to detect coronary artery disease in obese and nonobese patients. Journal of nuclear cardiology : official publication of the American Society of Nuclear Cardiology 2006;13:191-201.
68. Budoff MJ, Gillespie R, Georgiou D et al. Comparison of exercise electron beam computed tomography and sestamibi in the evaluation of coronary artery disease. The American journal of cardiology 1998;81:682-7.
69. Burns RJ, Galligan L, Wright LM, Lawand S, Burke RJ, Gladstone PJ. Improved specificity of myocardial thallium-201 single-photon emission computed tomography in patients with left bundle branch block by dipyridamole. The American journal of cardiology 1991;68:504-8.
70. Burns RJ, Iles S, Fung AY, Wright LM, Daigneault L. The Canadian exercise technetium 99m-labeled teboroxime single-photon emission computed tomographic study. Canadian Exercise Teboroxime SPECT Study Investigators. Journal of nuclear cardiology : official publication of the American Society of Nuclear Cardiology 1995;2:117-25.
71. Caobelli F, Akin M, Thackeray JT et al. Diagnostic accuracy of cadmium-zinc-telluride-based myocardial perfusion SPECT: impact of attenuation correction using a co-registered external computed tomography. European heart journal cardiovascular Imaging 2016;17:1036-43.
72. Cury RC, Kitt TM, Feaheny K et al. A randomized, multicenter, multivendor study of myocardial perfusion imaging with regadenoson CT perfusion vs single photon emission CT. Journal of cardiovascular computed tomography 2015;9:103-12.e1-2.
73. Candell-Riera J, Santana-Boado C, Castell-Conesa J et al. Simultaneous dipyridamole/maximal subjective exercise with 99mTc-MIBI SPECT: improved diagnostic yield in coronary artery disease. Journal of the American College of Cardiology 1997;29:531-6.
74. Caner B, Karanfil A, Uysal U et al. Effect of an additional atropine injection during dobutamine infusion for myocardial SPET. Nuclear medicine communications 1997;18:567-73.

75. Christian TF, Miller TD, Bailey KR, Gibbons RJ. Noninvasive identification of severe coronary artery disease using exercise tomographic thallium-201 imaging. The American journal of cardiology 1992;70:14-20.
76. Cramer MJ, Verzijlbergen JF, Niemeyer MG et al. 99Tcm-sestamibi SPECT with combined dipyridamole and exercise stress in coronary artery disease. Nuclear medicine communications 1994;15:554-9.
77. Danias PG, Roussakis A, Ioannidis JP. Diagnostic performance of coronary magnetic resonance angiography as compared against conventional X-ray angiography: a meta-analysis. Journal of the American College of Cardiology 2004;44:1867-76.
78. Danad I, Raijmakers PG, Driessen RS et al. Comparison of Coronary CT Angiography, SPECT, PET, and Hybrid Imaging for Diagnosis of Ischemic Heart Disease Determined by Fractional Flow Reserve. JAMA cardiology 2017;2:1100-1107.
79. Driessen RS, Danad I, Stuijfzand WJ et al. Comparison of Coronary Computed Tomography Angiography, Fractional Flow Reserve, and Perfusion Imaging for Ischemia Diagnosis. Journal of the American College of Cardiology 2019;73:161-173.
80. Di Bello V, Bellina CR, Gori E et al. Incremental diagnostic value of dobutamine stress echocardiography and dobutamine scintigraphy (technetium 99m-labeled sestamibi single-photon emission computed tomography) for assessment of presence and extent of coronary artery disease. Journal of nuclear cardiology : official publication of the American Society of Nuclear Cardiology 1996;3:212-20.
81. Elhendy A, Sozzi FB, Valkema R, van Domburg RT, Bax JJ, Roelandt JR. Dobutamine technetium-99m tetrofosmin SPECT imaging for the diagnosis of coronary artery disease in patients with limited exercise capacity. Journal of nuclear cardiology : official publication of the American Society of Nuclear Cardiology 2000;7:649-54.
82. Elhendy A, Sozzi FB, van Domburg RT et al. Accuracy of exercise stress technetium 99m sestamibi SPECT imaging in the evaluation of the extent and location of coronary artery disease in patients with an earlier myocardial infarction. Journal of nuclear cardiology : official publication of the American Society of Nuclear Cardiology 2000;7:432-8.
83. Elhendy A, van Domburg RT, Sozzi FB, Poldermans D, Bax JJ, Roelandt JR. Impact of hypertension on the accuracy of exercise stress myocardial perfusion imaging for the diagnosis of coronary artery disease. Heart (British Cardiac Society) 2001;85:655-61.
84. Ficaro EP, Fessler JA, Shreve PD, Kritzman JN, Rose PA, Corbett JR. Simultaneous transmission/emission myocardial perfusion tomography. Diagnostic accuracy of attenuation-corrected 99mTc-sestamibi single-photon emission computed tomography. Circulation 1996;93:463-73.
85. Fleming RM, Kirkeeide RL, Taegtmeyer H, Adyanthaya A, Cassidy DB, Goldstein RA. Comparison of technetium-99m teboroxime tomography with automated quantitative coronary arteriography and thallium-201 tomographic imaging. Journal of the American College of Cardiology 1991;17:1297-302.
86. Fragasso G, Lu C, Dabrowski P, Pagnotta P, Sheiban I, Chierchia SL. Comparison of stress/rest myocardial perfusion tomography, dipyridamole and dobutamine stress echocardiography for the detection of coronary disease in hypertensive patients with chest pain and positive exercise test. Journal of the American College of Cardiology 1999;34:441-7.

87. Fiechter M, Ghadri JR, Kuest SM et al. Nuclear myocardial perfusion imaging with a novel cadmium-zinc-telluride detector SPECT/CT device: first validation versus invasive coronary angiography. European journal of nuclear medicine and molecular imaging 2011;38:2025-30.

88. Gallowitsch HJ, Sykora J, Mikosch P et al. Attenuation-corrected thallium-201 single-photon emission tomography using a gadolinium-153 moving line source: clinical value and the impact of attenuation correction on the extent and severity of perfusion abnormalities. European journal of nuclear medicine 1998;25:220-8.

89. Gentile R, Vitarelli A, Schillaci O et al. Diagnostic accuracy and prognostic implications of stress testing for coronary artery disease in the elderly. Ital Heart J 2001;2:539-45.

90. Genovesi D, Giorgetti A, Gimelli A et al. Impact of attenuation correction and gated acquisition in SPECT myocardial perfusion imaging: results of the multicentre SPAG (SPECT Attenuation Correction vs Gated) study. European journal of nuclear medicine and molecular imaging 2011;38:1890-8.

91. Go RT, Marwick TH, MacIntyre WJ et al. A prospective comparison of rubidium-82 PET and thallium-201 SPECT myocardial perfusion imaging utilizing a single dipyridamole stress in the diagnosis of coronary artery disease. Journal of nuclear medicine : official publication, Society of Nuclear Medicine 1990;31:1899-905.

92. Groutars RG, Verzijlbergen JF, Tiel-van Buul MM et al. The accuracy of 1-day dual-isotope myocardial SPECT in a population with high prevalence of coronary artery disease. The international journal of cardiovascular imaging 2003;19:229-38.

93. Gunalp B, Dokumaci B, Uyan C et al. Value of dobutamine technetium-99m-sestamibi SPECT and echocardiography in the detection of coronary artery disease compared with coronary angiography. Journal of nuclear medicine : official publication, Society of Nuclear Medicine 1993;34:889-94.

94. Hacot JP, Bojovic M, Delonca J, Meier B, Righetti A. Comparison of planar imaging and single-photon emission computed tomography for the detection and localization of coronary artery disease. Int J Card Imaging 1993;9:113-9.

95. Hambye AS, Vervaet A, Lieber S, Ranquin R. Diagnostic value and incremental contribution of bicycle exercise, first-pass radionuclide angiography, and 99mTc-labeled sestamibi single-photon emission computed tomography in the identification of coronary artery disease in patients without infarction. Journal of nuclear cardiology : official publication of the American Society of Nuclear Cardiology 1996;3:464-74.

96. Hays JT, Mahmarian JJ, Cochran AJ, Verani MS. Dobutamine thallium-201 tomography for evaluating patients with suspected coronary artery disease unable to undergo exercise or vasodilator pharmacologic stress testing. Journal of the American College of Cardiology 1993;21:1583-90.

97. He ZX, Iskandrian AS, Gupta NC, Verani MS. Assessing coronary artery disease with dipyridamole technetium-99m-tetrofosmin SPECT: a multicenter trial. Journal of nuclear medicine : official publication, Society of Nuclear Medicine 1997;38:44-8.

98. Hecht HS, DeBord L, Shaw R et al. Supine bicycle stress echocardiography versus tomographic thallium-201 exercise imaging for the detection of coronary artery disease. Journal of the American Society of Echocardiography : official publication of the American Society of Echocardiography 1993;6:177-85.

99. Heiba SI, Hayat NJ, Salman HS et al. Technetium-99m-MIBI myocardial SPECT: supine versus right lateral imaging and comparison with coronary arteriography. Journal of nuclear medicine : official publication, Society of Nuclear Medicine 1997;38:1510-4.
100. Hendel RC, Berman DS, Cullom SJ et al. Multicenter clinical trial to evaluate the efficacy of correction for photon attenuation and scatter in SPECT myocardial perfusion imaging. Circulation 1999;99:2742-9.
101. Ho FM, Huang PJ, Liau CS et al. Dobutamine stress echocardiography compared with dipyridamole thallium-201 single-photon emission computed tomography in detecting coronary artery disease. European heart journal 1995;16:570-5.
102. Ho YL, Wu CC, Huang PJ et al. Dobutamine stress echocardiography compared with exercise thallium-201 single-photon emission computed tomography in detecting coronary artery disease-effect of exercise level on accuracy. Cardiology 1997;88:379-85.
103. Ho YL, Wu CC, Huang PJ et al. Assessment of coronary artery disease in women by dobutamine stress echocardiography: comparison with stress thallium-201 single-photon emission computed tomography and exercise electrocardiography. American heart journal 1998;135:655-62.
104. Hoffmann R, Lethen H, Kleinhans E, Weiss M, Flachskampf FA, Hanrath P. Comparative evaluation of bicycle and dobutamine stress echocardiography with perfusion scintigraphy and bicycle electrocardiogram for identification of coronary artery disease. The American journal of cardiology 1993;72:555-9.
105. Huang PJ, Ho YL, Wu CC et al. Simultaneous dobutamine stress echocardiography and thallium-201 perfusion imaging for the detection of coronary artery disease. Cardiology 1997;88:556-62.
106. Huang PJ, Yen RF, Chieng PU, Chen ML, Su CT. Do beta-blockers affect the diagnostic sensitivity of dobutamine stress thallium-201 single photon emission computed tomographic imaging? Journal of nuclear cardiology : official publication of the American Society of Nuclear Cardiology 1998;5:34-9.
107. Iftikhar I, Koutelou M, Mahmarian JJ, Verani MS. Simultaneous perfusion tomography and radionuclide angiography during dobutamine stress. Journal of nuclear medicine : official publication, Society of Nuclear Medicine 1996;37:1306-10.
108. Iskandrian AS, Heo J, Nguyen T et al. Assessment of coronary artery disease using single-photon emission computed tomography with thallium-201 during adenosine-induced coronary hyperemia. The American journal of cardiology 1991;67:1190-4.
109. Ito S, Endo A, Okada T et al. Comparison of CTAC and prone imaging for the detection of coronary artery disease using CZT SPECT. Annals of nuclear medicine 2017;31:629-635.
110. Jeetley P, Hickman M, Kamp O et al. Myocardial contrast echocardiography for the detection of coronary artery stenosis: a prospective multicenter study in comparison with single-photon emission computed tomography. Journal of the American College of Cardiology 2006;47:141-5.
111. Kang X, Berman DS, Lewin H et al. Comparative ability of myocardial perfusion single-photon emission computed tomography to detect coronary artery disease in patients with and without diabetes mellitus. American heart journal 1999;137:949-57.

112. Kapur A, Latus KA, Davies G et al. A comparison of three radionuclide myocardial perfusion tracers in clinical practice: the ROBUST study. European journal of nuclear medicine and molecular imaging 2002;29:1608-16.
113. Karavidas AI, Matsakas EP, Lazaros GA et al. Comparison of myocardial contrast echocardiography with SPECT in the evaluation of coronary artery disease in asymptomatic patients with LBBB. International journal of cardiology 2006;112:334-40.
114. Katayama T, Ogata N, Tsuruya Y. Diagnostic accuracy of supine and prone thallium-201 stress myocardial perfusion single-photon emission computed tomography to detect coronary artery disease in inferior wall of left ventricle. Annals of nuclear medicine 2008;22:317-21.
115. Kawai Y, Morita K, Nozaki Y, Ohkusa T, Sakurai M, Tamaki N. Diagnostic value of 123I-betamethyl-p-iodophenyl-pentadecanoic acid (BMIPP) single photon emission computed tomography (SPECT) in patients with chest pain. Comparison with rest-stress 99mTc-tetrofosmin SPECT and coronary angiography. Circulation journal : official journal of the Japanese Circulation Society 2004;68:547-52.
116. Ker WDS, Neves DGD, Magalhães TA, Santos A, Mesquita CT, Nacif MS. Myocardial Perfusion by Coronary Computed Tomography in the Evaluation of Myocardial Ischemia: Simultaneous Stress Protocol with SPECT. Arquivos brasileiros de cardiologia 2019;113:1092-1101.
117. Kiat H, Van Train KF, Maddahi J et al. Development and prospective application of quantitative 2-day stress-rest Tc-99m methoxy isobutyl isonitrile SPECT for the diagnosis of coronary artery disease. American heart journal 1990;120:1255-66.
118. Kisacik HL, Ozdemir K, Altinyay E et al. Comparison of exercise stress testing with simultaneous dobutamine stress echocardiography and technetium-99m isonitrile single-photon emission computerized tomography for diagnosis of coronary artery disease. European heart journal 1996;17:113-9.
119. Korosoglou G, Dubart AE, DaSilva KG, Jr. et al. Real-time myocardial perfusion imaging for pharmacologic stress testing: added value to single photon emission computed tomography. American heart journal 2006;151:131-8.
120. Kupari M, Virtanen KS, Turto H et al. Exclusion of coronary artery disease by exercise thallium-201 tomography in patients with aortic valve stenosis. The American journal of cardiology 1992;70:635-40.
121. Li JM, Li T, Shi RF, Zhang LR. Comparative Analysis between SPECT Myocardial Perfusion Imaging and CT Coronary Angiography for Diagnosis of Coronary Artery Disease. International journal of molecular imaging 2012;2012:253475.
122. Lipiec P, Wejner-Mik P, Krzeminska-Pakula M et al. Accelerated stress real-time myocardial contrast echocardiography for the detection of coronary artery disease: comparison with 99mTc single photon emission computed tomography. Journal of the American Society of Echocardiography : official publication of the American Society of Echocardiography 2008;21:941-7.
123. Liu YB, Huang PJ, Su CT, Chieng PU, Wu CC, Ho YL. Comparison of S-T segment/heart rate slope with exercise thallium imaging and conventional S-T segment criteria in detecting coronary artery disease: effect of exercise level on accuracy. Cardiology 1998;89:229-34.
124. Mahmarian JJ, Pratt CM, Cocanougher MK, Verani MS. Altered myocardial perfusion in patients with angina pectoris or silent ischemia during exercise as assessed by quantitative thallium-201 single-photon emission computed tomography. Circulation 1990;82:1305-15.

125. Mairesse GH, Marwick TH, Vanoverschelde JL et al. How accurate is dobutamine stress electrocardiography for detection of coronary artery disease? Comparison with two-dimensional echocardiography and technetium-99m methoxyl isobutyl isonitrile (mibi) perfusion scintigraphy. Journal of the American College of Cardiology 1994;24:920-7.

126. Mak KH, Ang ES, Goh AS, Na KX, Sundram FX, Tan AT. Myocardial perfusion imaging with technetium-99m sestamibi SPECT in the evaluation of coronary artery disease. Australas Radiol 1995;39:112-7.

127. Marwick T, D'Hondt AM, Baudhuin T et al. Optimal use of dobutamine stress for the detection and evaluation of coronary artery disease: combination with echocardiography or scintigraphy, or both? Journal of the American College of Cardiology 1993;22:159-67.

128. Marwick TH, D'Hondt AM, Mairesse GH et al. Comparative ability of dobutamine and exercise stress in inducing myocardial ischaemia in active patients. Br Heart J 1994;72:31-8.

129. Matsumoto N, Sato Y, Suzuki Y et al. Usefulness of rapid low-dose/high-dose 1-day 99mTc-sestamibi ECG-gated myocardial perfusion single-photon emission computed tomography. Circulation journal : official journal of the Japanese Circulation Society 2006;70:1585-9.

130. Matzer L, Kiat H, Wang FP et al. Pharmacologic stress dual-isotope myocardial perfusion single-photon emission computed tomography. American heart journal 1994;128:1067-76.

131. McClellan JR, Dugan TM, Heller GV. Patterns of use and clinical utility of exercise thallium-201 single photon emission-computed tomography in a community hospital. Cardiology 1996;87:134-40.

132. Meyer M, Nance JW, Jr., Schoepf UJ et al. Cost-effectiveness of substituting dual-energy CT for SPECT in the assessment of myocardial perfusion for the workup of coronary artery disease. European journal of radiology 2012;81:3719-25.

133. Miller DD, Younis LT, Chaitman BR, Stratmann H. Diagnostic accuracy of dipyridamole technetium 99m-labeled sestamibi myocardial tomography for detection of coronary artery disease. Journal of nuclear cardiology : official publication of the American Society of Nuclear Cardiology 1997;4:18-24.

134. Miller TD, Hodge DO, Christian TF, Milavetz JJ, Bailey KR, Gibbons RJ. Effects of adjustment for referral bias on the sensitivity and specificity of single photon emission computed tomography for the diagnosis of coronary artery disease. Am J Med 2002;112:290-7.

135. Minoves M, Garcia A, Magrina J, Pavia J, Herranz R, Setoain J. Evaluation of myocardial perfusion defects by means of "bull's eye" images. Clinical cardiology 1993;16:16-22.

136. Nallamothu N, Ghods M, Heo J, Iskandrian AS. Comparison of thallium-201 single-photon emission computed tomography and electrocardiographic response during exercise in patients with normal rest electrocardiographic results. Journal of the American College of Cardiology 1995;25:830-6.

137. Neglia D, Rovai D, Caselli C et al. Detection of significant coronary artery disease by noninvasive anatomical and functional imaging. Circulation Cardiovascular imaging 2015;8.

138. Nishida C, Okajima K, Kudo T, Yamamoto T, Hattori R, Nishimura Y. The relationship between coronary artery calcification detected by non-gated multi-detector CT in patients with suspected ischemic heart disease and myocardial ischemia detected by thallium exercise stress testing. Annals of nuclear medicine 2005;19:647-53.
139. Nishimura S, Mahmarian JJ, Boyce TM, Verani MS. Quantitative thallium-201 single-photon emission computed tomography during maximal pharmacologic coronary vasodilation with adenosine for assessing coronary artery disease. Journal of the American College of Cardiology 1991;18:736-45.
140. Nguyen T, Heo J, Ogilby JD, Iskandrian AS. Single photon emission computed tomography with thallium-201 during adenosine-induced coronary hyperemia: correlation with coronary arteriography, exercise thallium imaging and two-dimensional echocardiography. Journal of the American College of Cardiology 1990;16:1375-83.
141. Ogilby JD, Iskandrian AS, Untereker WJ, Heo J, Nguyen TN, Mercuro J. Effect of intravenous adenosine infusion on myocardial perfusion and function. Hemodynamic/angiographic and scintigraphic study. Circulation 1992;86:887-95.
142. Oguzhan A, Kisacik HL, Ozdemir K et al. Comparison of exercise stress testing with dobutamine stress echocardiography and exercise technetium-99m isonitrile single photon emission computerized tomography for diagnosis of coronary artery disease. Jpn Heart J 1997;38:333-44.
143. Palmas W, Friedman JD, Diamond GA, Silber H, Kiat H, Berman DS. Incremental value of simultaneous assessment of myocardial function and perfusion with technetium-99m sestamibi for prediction of extent of coronary artery disease. Journal of the American College of Cardiology 1995;25:1024-31.
144. Pavlovic S, Sobic-Saranovic D, Djordjevic-Dikic A et al. Comparative utility of gated myocardial perfusion imaging and transthoracic coronary flow reserve for the assessment of coronary artery disease in patients with left bundle branch block. Nuclear medicine communications 2010;31:334-40.
145. Peltier M, Vancraeynest D, Pasquet A et al. Assessment of the physiologic significance of coronary disease with dipyridamole real-time myocardial contrast echocardiography. Comparison with technetium-99m sestamibi single-photon emission computed tomography and quantitative coronary angiography. Journal of the American College of Cardiology 2004;43:257-64.
146. Pennell DJ, Underwood SR, Swanton RH, Walker JM, Ell PJ. Dobutamine thallium myocardial perfusion tomography. Journal of the American College of Cardiology 1991;18:1471-9.
147. Pennell DJ, Mavrogeni SI, Forbat SM, Karwatowski SP, Underwood SR. Adenosine combined with dynamic exercise for myocardial perfusion imaging. Journal of the American College of Cardiology 1995;25:1300-9.
148. Płachcińska A, Włodarczyk M, Kovacevic-Kuśmierek K et al. Diagnostic performance of myocardial perfusion single-photon emission computed tomography with attenuation correction. Kardiologia polska 2016;74:32-39.
149. Pozzoli MM, Fioretti PM, Salustri A, Reijs AE, Roelandt JR. Exercise echocardiography and technetium-99m MIBI single-photon emission computed tomography in the detection of coronary artery disease. The American journal of cardiology 1991;67:350-5.

150. Quinones MA, Verani MS, Haichin RM, Mahmarian JJ, Suarez J, Zoghbi WA. Exercise echocardiography versus 201Tl single-photon emission computed tomography in evaluation of coronary artery disease. Analysis of 292 patients. Circulation 1992;85:1026-31.
151. Salustri A, Pozzoli MM, Hermans W et al. Relationship between exercise echocardiography and perfusion single-photon emission computed tomography in patients with single-vessel coronary artery disease. American heart journal 1992;124:75-83.
152. San Roman JA, Vilacosta I, Castillo JA et al. Selection of the optimal stress test for the diagnosis of coronary artery disease. Heart (British Cardiac Society) 1998;80:370-6.
153. Sandler MP, Videlefsky S, Delbeke D et al. Evaluation of myocardial ischemia using a rest metabolism/stress perfusion protocol with fluorine-18 deoxyglucose/technetium-99m MIBI and dual-isotope simultaneous-acquisition single-photon emission computed tomography. Journal of the American College of Cardiology 1995;26:870-8.
154. Santoro GM, Sciagra R, Buonamici P et al. Head-to-head comparison of exercise stress testing, pharmacologic stress echocardiography, and perfusion tomography as first-line examination for chest pain in patients without history of coronary artery disease. Journal of nuclear cardiology : official publication of the American Society of Nuclear Cardiology 1998;5:19-27.
155. Sand NPR, Veien KT, Nielsen SS et al. Prospective Comparison of FFR Derived From Coronary CT Angiography With SPECT Perfusion Imaging in Stable Coronary Artery Disease: The ReASSESS Study. JACC Cardiovascular imaging 2018;11:1640-1650.
156. Schaap J, Kauling RM, Boekholdt SM et al. Incremental diagnostic accuracy of hybrid SPECT/CT coronary angiography in a population with an intermediate to high pre-test likelihood of coronary artery disease. European heart journal cardiovascular Imaging 2013;14:642-9.
157. Schwartz JG, Johnson RB, Aepfelbacher FC et al. Sensitivity, specificity and accuracy of stress SPECT myocardial perfusion imaging for detection of coronary artery disease in the distribution of first-order branch vessels, using an anatomical matching of angiographic and perfusion data. Nuclear medicine communications 2003;24:543-9.
158. Senior R, Lepper W, Pasquet A et al. Myocardial perfusion assessment in patients with medium probability of coronary artery disease and no prior myocardial infarction: comparison of myocardial contrast echocardiography with 99mTc single-photon emission computed tomography. American heart journal 2004;147:1100-5.
159. Sharir T, Bacher-Stier C, Dhar S et al. Identification of severe and extensive coronary artery disease by postexercise regional wall motion abnormalities in Tc-99m sestamibi gated single-photon emission computed tomography. The American journal of cardiology 2000;86:1171-5.
160. Smart SC, Bhatia A, Hellman R et al. Dobutamine-atropine stress echocardiography and dipyridamole sestamibi scintigraphy for the detection of coronary artery disease: limitations and concordance. Journal of the American College of Cardiology 2000;36:1265-73.
161. Solot G, Hermans J, Merlo P et al. Correlation of 99Tcm-sestamibi SPECT with coronary angiography in general hospital practice. Nuclear medicine communications 1993;14:23-9.

162. Soman P, Khattar R, Senior R, Lahiri A. Inotropic stress with arbutamine is superior to vasodilator stress with dipyridamole for the detection of reversible ischemia with Tc-99m sestamibi single-photon emission computed tomography. Journal of nuclear cardiology : official publication of the American Society of Nuclear Cardiology 1997;4:364-71.
163. Squires SR, Bushnell DL, Menda Y, Graham MM. Comparison of cardiac to hepatic uptake of 99mTc-tetrofosmin with and without adenosine infusion to predict the presence of haemodynamically significant coronary artery disease. Nuclear medicine communications 2005;26:513-8.
164. Stewart RE, Schwaiger M, Molina E et al. Comparison of rubidium-82 positron emission tomography and thallium-201 SPECT imaging for detection of coronary artery disease. The American journal of cardiology 1991;67:1303-10.
165. Sylven C, Hagerman I, Ylen M, Nyquist O, Nowak J. Variance ECG detection of coronary artery disease--a comparison with exercise stress test and myocardial scintigraphy. Clinical cardiology 1994;17:132-40.
166. Tadehara F, Yamamoto H, Tsujiyama S et al. Feasibility of a rapid protocol of 1-day single-isotope rest/adenosine stress Tc-99m sestamibi ECG-gated myocardial perfusion imaging. Journal of nuclear cardiology : official publication of the American Society of Nuclear Cardiology 2008;15:35-41.
167. Taillefer R, DePuey EG, Udelson JE, Beller GA, Latour Y, Reeves F. Comparative diagnostic accuracy of Tl-201 and Tc-99m sestamibi SPECT imaging (perfusion and ECG-gated SPECT) in detecting coronary artery disease in women. Journal of the American College of Cardiology 1997;29:69-77.
168. Takeishi Y, Sukekawa H, Saito H et al. Left ventricular function and myocardial perfusion during dipyridamole infusion assessed by a single injection of 99Tcm-sestamibi in patients unable to exercise. Nuclear medicine communications 1994;15:697-703.
169. Takeuchi M, Araki M, Nakashima Y, Kuroiwa A. Comparison of dobutamine stress echocardiography and stress thallium-201 single-photon emission computed tomography for detecting coronary artery disease. Journal of the American Society of Echocardiography : official publication of the American Society of Echocardiography 1993;6:593-602.
170. Tartagni F, Dondi M, Limonetti P et al. Dipyridamole technetium-99m-2-methoxy isobutyl isonitrile tomoscintigraphic imaging for identifying diseased coronary vessels: comparison with thallium-201 stress-rest study. Journal of nuclear medicine : official publication, Society of Nuclear Medicine 1991;32:369-76.
171. Travin MI, Katz MS, Moulton AW, Miele NJ, Sharaf BL, Johnson LL. Accuracy of dipyridamole SPECT imaging in identifying individual coronary stenoses and multivessel disease in women versus men. Journal of nuclear cardiology : official publication of the American Society of Nuclear Cardiology 2000;7:213-20.
172. Tsai MF, Kao PF, Tzen KY. Improved diagnostic performance of thallium-201 myocardial perfusion scintigraphy in coronary artery disease: from planar to single photon emission computed tomography imaging. Chang Gung Med J 2002;25:522-30.
173. Tsutsui JM, Xie F, McGrain AC et al. Comparison of low-mechanical index pulse sequence schemes for detecting myocardial perfusion abnormalities during vasodilator stress echocardiography. The American journal of cardiology 2005;95:565-70.

174. van Diemen PA, Driessen RS, Kooistra RA et al. Comparison Between the Performance of Quantitative Flow Ratio and Perfusion Imaging for Diagnosing Myocardial Ischemia. JACC Cardiovascular imaging 2020.

175. Van Train KF, Maddahi J, Berman DS et al. Quantitative analysis of tomographic stress thallium-201 myocardial scintigrams: a multicenter trial. Journal of nuclear medicine : official publication, Society of Nuclear Medicine 1990;31:1168-79.

176. Van Train KF, Areeda J, Garcia EV et al. Quantitative same-day rest-stress technetium-99m-sestamibi SPECT: definition and validation of stress normal limits and criteria for abnormality. Journal of nuclear medicine : official publication, Society of Nuclear Medicine 1993;34:1494-502.

177. Van Train KF, Garcia EV, Maddahi J et al. Multicenter trial validation for quantitative analysis of same-day rest-stress technetium-99m-sestamibi myocardial tomograms. Journal of nuclear medicine : official publication, Society of Nuclear Medicine 1994;35:609-18.

178. Verani MS, Mahmarian JJ, Hixson JB, Boyce TM, Staudacher RA. Diagnosis of coronary artery disease by controlled coronary vasodilation with adenosine and thallium-201 scintigraphy in patients unable to exercise. Circulation 1990;82:80-7.

179. Wang FP, Amanullah AM, Kiat H, Friedman JD, Berman DS. Diagnostic efficacy of stress technetium 99m-labeled sestamibi myocardial perfusion single-photon emission computed tomography in detection of coronary artery disease among patients over age 80. Journal of nuclear cardiology : official publication of the American Society of Nuclear Cardiology 1995;2:380-8.

180. Watanabe K, Sekiya M, Ikeda S, Miyagawa M, Kinoshita M, Kumano S. Comparison of adenosine triphosphate and dipyridamole in diagnosis by thallium-201 myocardial scintigraphy. Journal of nuclear medicine : official publication, Society of Nuclear Medicine 1997;38:577-81.

181. Weinsaft JW, Gade CL, Wong FJ et al. Diagnostic impact of SPECT image display on assessment of obstructive coronary artery disease. Journal of nuclear cardiology : official publication of the American Society of Nuclear Cardiology 2007;14:659-68.

182. Wu MC, Chin KC, Lin KH, Chiu NT. Diagnostic efficacy of a low-dose 32-projection SPECT 99mTc-sestamibi myocardial perfusion imaging protocol in routine practice. Nuclear medicine communications 2009;30:140-7.

183. Xin W, Yang X, Wang J et al. Gated single-photon emission computed tomography myocardial perfusion imaging is superior to computed tomography attenuation correction in discriminating myocardial infarction from attenuation artifacts in men and right coronary artery disease. Nuclear medicine communications 2019;40:491-498.

184. Yeih DF, Huang PJ, Ho YL. Enhanced diagnosis of coronary artery disease in women by dobutamine thallium-201 ST-segment/heart rate slope and thallium-201 myocardial SPECT. J Formos Med Assoc 2007;106:832-9.

185. Aggarwal NR, Drozdova A, Askew JW, 3rd, Kemp BJ, Chareonthaitawee P. Feasibility and diagnostic accuracy of exercise treadmill nitrogen-13 ammonia PET myocardial perfusion imaging of obese patients. Journal of nuclear cardiology : official publication of the American Society of Nuclear Cardiology 2015;22:1273-80.

186. Bateman TM, Heller GV, McGhie AI et al. Diagnostic accuracy of rest/stress ECG-gated Rb-82 myocardial perfusion PET: comparison with ECG-gated Tc-99m sestamibi SPECT. Journal of nuclear cardiology : official publication of the American Society of Nuclear Cardiology 2006;13:24-33.
187. Botsch H, Beringer K, Petersen J, Bauer B, Weidemann H. Single-photon emission tomography studies of rubidium-81 in the detection of ischaemic heart disease, using a stress-reinjection protocol. European journal of nuclear medicine 1994;21:407-14.
188. Chow BJ, Dennie C, Hoffmann U et al. Comparison of computed tomographic angiography versus rubidium-82 positron emission tomography for the detection of patients with anatomical coronary artery disease. The Canadian journal of cardiology 2007;23:801-7.
189. Dekker M, Waissi F, Bank IEM et al. Automated calcium scores collected during myocardial perfusion imaging improve identification of obstructive coronary artery disease. International journal of cardiology Heart & vasculature 2020;26:100434.
190. Esteves FP, Sanyal R, Nye JA, Santana CA, Verdes L, Raggi P. Adenosine stress rubidium-82 PET/computed tomography in patients with known and suspected coronary artery disease. Nuclear medicine communications 2008;29:674-8.
191. Fathala A, Aboulkheir M, Shoukri MM, Alsergani H. Diagnostic accuracy of (13)N-ammonia myocardial perfusion imaging with PET-CT in the detection of coronary artery disease. Cardiovascular diagnosis and therapy 2019;9:35-42.
192. Grover-McKay M, Ratib O, Schwaiger M et al. Detection of coronary artery disease with positron emission tomography and rubidium 82. American heart journal 1992;123:646-52.
193. Kaster T, Mylonas I, Renaud JM, Wells GA, Beanlands RS, deKemp RA. Accuracy of low-dose rubidium-82 myocardial perfusion imaging for detection of coronary artery disease using 3D PET and normal database interpretation. Journal of nuclear cardiology : official publication of the American Society of Nuclear Cardiology 2012;19:1135-45.
194. Laubenbacher C, Rothley J, Sitomer J et al. An automated analysis program for the evaluation of cardiac PET studies: initial results in the detection and localization of coronary artery disease using nitrogen-13-ammonia. Journal of nuclear medicine : official publication, Society of Nuclear Medicine 1993;34:968-78.
195. Lee JM, Kim CH, Koo BK et al. Integrated Myocardial Perfusion Imaging Diagnostics Improve Detection of Functionally Significant Coronary Artery Stenosis by 13N-ammonia Positron Emission Tomography. Circulation Cardiovascular imaging 2016;9.
196. Marwick TH, Nemec JJ, Stewart WJ, Salcedo EE. Diagnosis of coronary artery disease using exercise echocardiography and positron emission tomography: comparison and analysis of discrepant results. Journal of the American Society of Echocardiography : official publication of the American Society of Echocardiography 1992;5:231-8.
197. Sampson UK, Dorbala S, Limaye A, Kwong R, Di Carli MF. Diagnostic accuracy of rubidium-82 myocardial perfusion imaging with hybrid positron emission tomography/computed tomography in the detection of coronary artery disease. Journal of the American College of Cardiology 2007;49:1052-8.

198. Santana CA, Folks RD, Garcia EV et al. Quantitative (82)Rb PET/CT: development and validation of myocardial perfusion database. Journal of nuclear medicine : official publication, Society of Nuclear Medicine 2007;48:1122-8.
199. Shi H, Santana CA, Rivero A et al. Normal values and prospective validation of transient ischaemic dilation index in 82Rb PET myocardial perfusion imaging. Nuclear medicine communications 2007;28:859-63.
200. Simone GL, Mullani NA, Page DA, Anderson BA, Sr. Utilization statistics and diagnostic accuracy of a nonhospital-based positron emission tomography center for the detection of coronary artery disease using rubidium-82. Am J Physiol Imaging 1992;7:203-9.
201. Strähl M, Schindler M, Keller LS et al. Diagnostic performance of angiography-based quantitative flow ratio for the identification of myocardial ischemia as assessed by (13)N-ammonia myocardial perfusion imaging positron emission tomography. International journal of cardiology 2020;314:13-19.
202. Wallhaus TR, Lacy J, Stewart R et al. Copper-62-pyruvaldehyde bis(N-methyl-thiosemicarbazone) PET imaging in the detection of coronary artery disease in humans. Journal of nuclear cardiology : official publication of the American Society of Nuclear Cardiology 2001;8:67-74.
203. Williams BR, Mullani NA, Jansen DE, Anderson BA. A retrospective study of the diagnostic accuracy of a community hospital-based PET center for the detection of coronary artery disease using rubidium-82. Journal of nuclear medicine : official publication, Society of Nuclear Medicine 1994;35:1586-92.
